# A dominant-negative SOX18 mutant disrupts multiple regulatory layers essential to transcription factor activity

**DOI:** 10.1093/nar/gkab820

**Published:** 2021-09-27

**Authors:** Alex J McCann, Jieqiong Lou, Mehdi Moustaqil, Matthew S Graus, Ailisa Blum, Frank Fontaine, Hui Liu, Winnie Luu, Paulina Rudolffi-Soto, Peter Koopman, Emma Sierecki, Yann Gambin, Frédéric A Meunier, Zhe Liu, Elizabeth Hinde, Mathias Francois

**Affiliations:** Institute for Molecular Bioscience, The University of Queensland, Brisbane, QLD 4072, Australia; School of Physics, Department of Biochemistry and Molecular Biology, Bio21, The University of Melbourne, Melbourne, VIC 3010, Australia; EMBL Australia Node in Single Molecule Science and School of Medical Sciences, The University of New South Wales, Sydney, NSW 1466, Australia; The David Richmond Laboratory for Cardio-Vascular Development: gene regulation and editing, The Centenary Institute, Newtown, Sydney, NSW 2006, Australia; Clem Jones Centre for Ageing Dementia Research, Queensland Brain Institute, The University of Queensland, Brisbane, QLD 4072, Australia; Institute for Molecular Bioscience, The University of Queensland, Brisbane, QLD 4072, Australia; Janelia Research Campus, Howard Hughes Medical Institute, Ashburn, VA 20147, United States; The David Richmond Laboratory for Cardio-Vascular Development: gene regulation and editing, The Centenary Institute, Newtown, Sydney, NSW 2006, Australia; EMBL Australia Node in Single Molecule Science and School of Medical Sciences, The University of New South Wales, Sydney, NSW 1466, Australia; Institute for Molecular Bioscience, The University of Queensland, Brisbane, QLD 4072, Australia; EMBL Australia Node in Single Molecule Science and School of Medical Sciences, The University of New South Wales, Sydney, NSW 1466, Australia; EMBL Australia Node in Single Molecule Science and School of Medical Sciences, The University of New South Wales, Sydney, NSW 1466, Australia; Clem Jones Centre for Ageing Dementia Research, Queensland Brain Institute, The University of Queensland, Brisbane, QLD 4072, Australia; Janelia Research Campus, Howard Hughes Medical Institute, Ashburn, VA 20147, United States; School of Physics, Department of Biochemistry and Molecular Biology, Bio21, The University of Melbourne, Melbourne, VIC 3010, Australia; Institute for Molecular Bioscience, The University of Queensland, Brisbane, QLD 4072, Australia; The David Richmond Laboratory for Cardio-Vascular Development: gene regulation and editing, The Centenary Institute, Newtown, Sydney, NSW 2006, Australia; School of Life and Environmental Sciences, The University of Sydney, Sydney, NSW 2006, Australia

## Abstract

Few genetically dominant mutations involved in human disease have been fully explained at the molecular level. In cases where the mutant gene encodes a transcription factor, the dominant-negative mode of action of the mutant protein is particularly poorly understood. Here, we studied the genome-wide mechanism underlying a dominant-negative form of the SOX18 transcription factor (SOX18^RaOp^) responsible for both the classical mouse mutant Ragged Opossum and the human genetic disorder Hypotrichosis-lymphedema-telangiectasia-renal defect syndrome. Combining three single-molecule imaging assays in living cells together with genomics and proteomics analysis, we found that SOX18^RaOp^ disrupts the system through an accumulation of molecular interferences which impair several functional properties of the wild-type SOX18 protein, including its target gene selection process. The dominant-negative effect is further amplified by poisoning the interactome of its wild-type counterpart, which perturbs regulatory nodes such as SOX7 and MEF2C. Our findings explain in unprecedented detail the multi-layered process that underpins the molecular aetiology of dominant-negative transcription factor function.

## INTRODUCTION

Embryonic development is dependent upon the activity of transcription factor (TF) complexes, which assemble on the chromatin in a finely orchestrated temporal and spatial sequence to coordinate the expression of specific gene programs (1,2). A current challenge in the study of human genetic disease is to understand how perturbed TF function leads to the phenotypic spectrum at the molecular level. For recessive disorders, loss or impairment of TF function provides a ready explanation. However, dominant disorders have been more difficult to pin down, but are ascribed to dominant-negative TF activity, whereby a mutant protein interferes with the functionality of its wild-type counterpart. Concepts such as neomorphism (where the mutated gene product takes on novel functions) and antimorphism (where the mutated gene product antagonizes the wild-type gene product) are commonly evoked (3). Despite this, it is currently not clear whether or how these concepts manifest at the molecular level; nor is it clear whether these are the only possible modes of action of dominant inheritance. Several classes of dominant-negative TF mutations have been described, including those causing truncation, deletion or alteration of either the DNA-binding domain or another functional domain (4). However, the key to understanding how these mutant TFs act in a dominant-negative fashion lies in discovering not only how the mutant protein lacks the function of the normal protein, but also how it actively interferes with the function of the normal protein (5).

TFs function primarily as heterodimers or homodimers. Where a dominant-negative TF is expressed, it has been proposed that the overall protein functionality of TFs that form homodimers will be only 25% of the wild-type, since of the four potential homodimer configurations (wt/wt, wt/mut, mut/wt, mut/mut) only one (wt/wt) is functional, with the mutant form effectively poisoning the other complexes (5). Moreover, over-expression of a dominant-negative allele would amplify the observed negative effects, bringing functionality to <25% (5). These scenarios potentially explain why dominant-negative mutations are usually more severe than recessive, loss-of-function mutations that would be predicted to reduce TF activity to ∼50% of the normal level.

Dominant-negative mutations causing genetic disorders have been observed to occur in genes encoding a number of TF in the SOX family (6–10). SOX TFs are key molecular switches of cell fate in numerous tissue types during embryogenesis (11). These include SOX8, 9 and 10 in which dominant-negative mutations trigger sex determination disorders (8), skeletal defects (9), and neural crest dysfunction (10) respectively.

Here, we have studied a dominant-negative mutation affecting the TF SOX18. Over the last 70 years, the study of SOX18 and its mutants in mice, humans and zebrafish have yielded profound insights into the regulation of vascular, lymphatic and hair follicle development (6,7,12–16), and into the concepts of allelic series, genetic redundancy and the mode of action of genetic modifiers (17,18). Despite an expression pattern suggesting an important role in vascular and hair follicle development (14), early attempts to understand the developmental role of SOX18 through the generation of Sox18-null mice were confounded by the surprisingly mild phenotype of normal vascular development with only mild hair follicle anomalies (19). SOX18 is co-expressed with closely related ‘SOXF’ subfamily members SOX7 and SOX17 in endothelial cells (17,20), suggesting that the lack of phenotype of Sox18-null mice is due to functional redundancy between the three TFs.

This phenotype contrasts with that of a classical mouse mutant, Ragged, found to result from nonsense mutations in Sox18 (14). Discovered in the 1950s (12,13), four Ragged alleles were described, the most severe of which is known as Ragged opossum (Sox18^RaOp^) (21–23). Inheritance of one Sox18^RaOp^ allele is sufficient to yield a thin, ragged coat and vascular leakage. The presence of two mutant alleles together causes death *in utero* due to lethal vascular dysfunction and/or lymphedema (24). The dramatically different phenotypes of Sox18-null and Sox18^RaOp^ mice suggested a dominant-negative mechanism of the latter, whereby SOX18^RaOp^ interferes with the function of SOX18 and also SOX7 and SOX17. While there has been some progress in elucidating the genetic pathways downstream of SOX18 (15,25–27), it remains unclear how the mutant SOX18^RaOp^ protein interferes with wild-type SOX18 function to perturb downstream gene expression.

Analogous to SOX18^RaOp^ mice, dominant mutations in SOX18 in humans cause the rare congenital disorder Hypotrichosis-lymphedema-telangiectasia-renal defect syndrome (HLTRS) (6,7). Patients diagnosed with HLTRS exhibit prominent hair follicle and vascular defects; hair follicles are sparse or absent, lymphatic vessels leak causing swollen limbs, and various vascular defects are present, including those that can cause renal failure. An unexplained etiological component of HLTRS is that SOX18 mutations, akin to mouse, occur in allelic series which underpin the severity of the syndrome with defects ranging from mild to lethal (28).

An understanding of how dominant-negative SOX18 proteins give rise to a range of phenotypic outcomes in SOX18^RaOp^ mice and HLTRS children rests on detailed knowledge of the mode of action of the wild-type SOX18 TF. Here, we apply a suite of molecular imaging assays to visualise SOX18 nuclear dynamics and analyse its search pattern on the chromatin. We use this pipeline to quantify dominant-negative effects of mutant SOX18 proteins beyond simple genetic configuration and level of allelic expression. We show that altered biophysical parameters such as nuclear concentration, diffusion, oligomeric state, chromatin-binding dynamics, chromatin-binding affinity, protein stability, protein partner recruitment and TF binding motif selection unbalance the regulatory network in favour of the mutant protein. This study defines novel mechanisms of interference that underlie dominant-negative TF action, providing new insights into the mechanisms that underpin dominant human genetic disorders.

## MATERIALS AND METHODS

For information regarding the software, algorithms and publicly available datasets used throughout this manuscript, please refer to Table 1.

**Table 1.** List of software, algorithms and publicly available datasets that were used in this manuscript

Software and algorithms	Source	Information
ZEISS ZEN blue	ZEISS Microscopy	https://www.zeiss.com/microscopy/int/products/microscope-software/zen.html
ImageJ 1.53c	National Institutes of Health	http://imagej.nih.gov/ij
MetaMorph v7.8.0.0	Molecular Devices	https://www.moleculardevices.com/products/cellular-imaging-systems/acquisition-and-analysis-software/metamorph-microscopy
PalmTracer MetaMorph plugin	Sibarita Group, University of Bordeaux Izeddin *et al.* (30)	PalmTracer is based off of WaveTracer in Izeddin *et al.*, 2012 (30)
AutoAnalysis_SPT	Developed by Dr Liz Cooper-Williams for the Meunier Group at the Queensland Brain Institute	https://github.com/QBI-Software/AutoAnalysis_SPT/wiki
MATLABR2015a	MathWorks Inc.	https://au.mathworks.com/products/matlab.html
SLIMfast MatLab script	Developed by Chen *et al.* (2) based on Multiple-Target Tracing (MTT) algorithms developed by Serge *et al.* (32)	Provided by Zhe Liu: liuz11@janelia.hhmi.org
DiffusionSingle MATLAB script	Developed by Chen *et al.* (2)	Provided by Zhe Liu: liuz11@janelia.hhmi.org
MSDanalyzer MATLAB script	Developed by Tarantino *et al.* (66)	https://au.mathworks.com/matlabcentral/fileexchange/40692-mean-square-displacement-analysis-of-particles-trajectories
Cas9DiffusionCombineAll MATLAB script		Provided by Zhe Liu: liuz11@janelia.hhmi.org
Calculatelength_2fitting_v3 MATLAB script		Provided by Zhe Liu: liuz11@janelia.hhmi.org
Spot-On MATLAB script	Developed by Hansen *et al.* (33)	https://gitlab.com/tjian-darzacq-lab/spot-on-matlab
Microsoft Excel	Microsoft	https://www.microsoft.com/en-au/microsoft-365/excel
GraphPad Prism v8.0	GraphPad Software	https://www.graphpad.com/scientific-software/prism/
EpiExplorer webtool	Developed by the Computational Epigenetics Group at the Max-Plank Institute for Informatics Halachev *et al.* (47)	https://epiexplorer.mpi-inf.mpg.de/
SimFCS	Laboratory for Fluorescence Dynamics	www.lfd.uci.edu
Expasy pI calculator	SIB Swiss Institute of Bioinformatics	https://www.expasy.org/resources/compute-pi-mw
MACS v2.1.0	Developed by Zhang *et al.* (67)	
BWA v0.7.12	Developed by Li and Durbin (68)	
bcl2fastq2 v0.1.19	Illumina	https://sapac.support.illumina.com/downloads/bcl2fastq-conversion-software-v2-20.html
BEDtools v2.25.0	Developed by Quinlan and Hall (69)	https://github.com/arq5x/bedtools2
wigToBigWig v4	ENCODE	https://www.encodeproject.org/software/wigtobigwig/
Data	Source	Information
SOX18-myc ChIP-seq data in HUVECs	Francois Group, Institute for Molecular Bioscience Overman *et al.* (34)	https://www.ebi.ac.uk/arrayexpress/experiments/E-MTAB-4481/
Histone marks and RNA polymerase II ChIP-seq data in HUVECs	ENCODE consortium	https://www.ebi.ac.uk/arrayexpress/experiments/E-GEOD-29611/

### Plasmid generation

A list of plasmids used in this study is provided in Supplementary Table S1. Protein expression for all plasmids, except luciferase constructs, is driven by a CMV promoter.

pReceiver-M49(HaloTag-SOX18) was obtained from GeneCopoeia, and subsequently used as a template to generate all other HaloTag constructs using the In-Fusion HD Cloning Kit (Takara Bio USA, Inc), with the exception of pReciever-M49(HaloTag-SOX17) which was also obtained from GeneCopoeia.

Alpha-helix 1 of the SOX HMG domain consisting of 18 amino acids (84–101) was removed from pReceiver-M49(HaloTag-SOX18) using a combination of circular polymerase extension cloning (CPEC) with In-Fusion cloning to generate the pReceiver-M49(HaloTag-SOX18ΔAH1) construct.

The homodimerization domain consisting of 45 amino acids (155–199) adjacent to the C-terminal NLS was removed from pReciever-M49(HaloTag-SOX18) using a combination of circular polymerase extension cloning (CPEC) with In-Fusion cloning to generate the pReceiver-M49(HaloTag-SOX18ΔDIM) construct.

The HaloTag was removed from pReceiver-M49(HaloTag-SOX18) and pReceiver-M49(HaloTag-SOX18RaOp) constructs using a combination of CPEC with In-Fusion cloning to generate the pReciever-M49(untagged-SOX18) and pReciever-M49(untagged-SOX18RaOp) constructs.

### Western blotting

Western blotting was used to assess the level of endogenous SOX18 protein in HeLa cells and HUVECs (Supplementary Figure S2A), and to compare the expression level and nuclear concentration of overexpressed HALO-SOX18 and HALO-SOX18^RaOp^ protein in HeLa cells (Supplementary Figure S2C). Cells were seeded, and either transfected (using 1 μg of expression plasmid to 3 μl of X-tremeGENE 9) or left untransfected, and harvested for either whole cell lysates or nuclear extracts before subjecting to SDS-PAGE and Western Blotting with a human anti-SOX18 antibody (sc166025 from Santa Cruz), anti-HaloTag antibody (G9281 from Promega), or housekeeping control anti-β-actin antibody (A5441 from Sigma).

### Luciferase assay – Dominant-negative effect of SOX18^RaOp^ on SOX18

Seven thousand monkey kidney fibroblast-like COS-7 cells were seeded per well in gelatin-coated 96-well plates (Gibco DMEM, Cat# 11995073, 10% v/v heat-inactivated foetal bovine serum, 1% l-glutamine, penicillin, streptomycin). Cells were maintained at 37°C, in a 5% CO_2_ controlled atmosphere. After 24 h, a 4-h transfection with murine plasmids, pGL2-Basic (Promega) VCAM-1 promoter construct (VC1889 (29); 40 ng/well), with and without HALO-SOX18 and/or untagged-SOX18^RaOp^ was performed. HALO-SOX18 alone = 30 ng/well, SOX18^RaOp^ alone = 10 ng/well, 30:1 ratio = 30 ng HALO-SOX18: 1 ng SOX18^RaOp^ etc., premix X-tremeGENE HP DNA transfection reagent was used in a 1:4 DNA:X-tremeGENE HP ratio (Roche/Sigma, Cat# 6366236001). A robust regression and outlier removal (ROUT) outlier test using default settings (*Q* = 1%) in GraphPad Prism (version 9.0.0) was performed to identify and remove outliers. Data was log transformed in order to meet the assumptions of homoscedasticity and normality of residuals required for ANOVA analysis. Pairwise comparisons were performed using a Tukey post-hoc test.

### Luciferase assay: dominant-negative effect of SOX18^RaOp^ on SOXF factors

HeLa cells were seeded in 24-well plates at 9 × 10^4^ cells/well in triplicate per condition. The cells were transfected with 250 ng of pGL3-basic or human VCAM1 promoter, with or without 40 ng of SOX18, SOX17, SOX7, and SOX18^RaOp^ for 24 h. Transfections were performed using the X-tremeGENE 9 Transfection Reagent kit (Roche) as per manufacturer's instructions, with a DNA to X-tremeGENE 9 ratio of 1 μg to 3 μl. After 24 h, the cells were washed twice in PBS and harvested using the luciferase assay reporter system, according to the manufacturer's instructions. Firefly luciferase activity was determined and normalised to protein concentration to control for cell number, pGL3-basic activity subtracted from the VCAM promoter activity, and then made relative to the +VCAM +SOX18 condition, which was set to 1. These values were plotted into GraphPad Prism (version 9.0.0), with the mean ± s.e.m. shown. Statistical significance for pairwise comparisons was assessed using ANOVA, multiple comparisons were corrected using the Šidák correction.

### Cell culture

HeLa cells were a gift from Professor Geoffrey Faulkner (Queensland Brain Institute/Translational Research Institute, Brisbane, QLD, Australia). Cells were cultured in Dulbecco's Modified Eagle Media (DMEM, Gibco) supplemented with 10% Fetal Bovine Serum (FBS, GE Healthcare), 1% GlutaMAX (Gibco) and 1% MEM non-essential amino acids (MEM NEAA, Gibco). Cells were maintained at 37°C with 5% CO_2_.

### Cell seeding and transfection

HeLa cells were seeded at a density of 155 000 cells in 29 mm (D29-20-1.5-N, Cellvis) or 35 mm (P35G-1.5-20-C, Matek) glass coverslip dishes coated with 1% gelatin 24 h prior to transfection. Transfections were performed using the X-tremeGENE 9 Transfection Reagent kit (Roche) to introduce 1–2 μg of plasmid DNA as per manufacturer's instructions, using FluoroBrite DMEM (Gibco) supplemented with 1% GlutaMAX (Gibco) as the low serum transfection media. Cells were incubated at 37°C with 5% CO_2_ for 24 h prior to imaging.

### Single molecule tracking: imaging

Immediately prior to imaging, cells were washed twice and replaced with FluoroBrite DMEM (Gibco) imaging media. JF549 dye was a gift from Dr Luke Lavis (Janelia Research Campus, Howard Hughes Medical Institute, Ashburn, VA, United States). 2 drops/ml of NucBlue Live ReadyProbes Reagent (Hoechst 33342) was added directly to the media and cells were incubated for 5 min at 37°C with 5% CO_2_, prior to adding 2 nM of JF549 Halo-tag dye directly to the media and cells were incubated for a further 15 min at 37°C with 5% of CO_2_. Following incubation, cells were washed twice and replaced with FluoroBrite DMEM (Gibco) containing 20 mM HEPES.

Images were acquired on an Elyra single molecule imaging (PALM/STORM) total internal reflection fluorescence (TIRF) microscope, with an Andor 897 EMCCD camera, SR Cube 05 RL – BP 420–480/BP 570–640/LP 740 filter set and 100× oil 1.46 NA TIRF objective using ZEISS ZEN blue software.

Cells were imaged using a 561 nm excitation laser (power = 11.6 μW oblique illumination, 1.96 mW epi illumination; power density = 0.728 W/cm^2^ oblique illumination, 123.24 W/cm^2^ epi illumination) with a high-power TIRF filter (TIRF_HP). The laser power was measured using a power meter at the level of the objective in oblique illumination and epi illumination. The power density (*I*) was calculated using Equation (1):

(1) $$\begin{equation*}I = \left( {\frac{P}{{\pi {r^2}}}} \right)\;\times\;4\end{equation*}$$

where *P* is power in watts (11.6 × 10^–6^ W for oblique illumination and 1.96 × 10^–3^ W for epi illumination), and *r* is the radius of the area being illuminated in cm (45 × 10^–4^ cm), which was measured by burning a spot on a dye specimen using the 100× oil objective and measuring the diameter of the spot using the 10× air objective (90 μm). The power density is multiplied by 4 as the TIRF_HP filter produces a power density that is approximately ∼4× higher compared to TIRF. Using these parameters, we performed two different acquisition techniques; fast SMT which uses a 20 ms acquisition speed to acquire 6000 frames without intervals, and slow SMT which uses a 500 ms acquisition speed to acquire 500 frames without intervals. A low laser power was used to achieve a good signal-to-noise ratio with minimal photobleaching during imaging. Target and surrounding cells were prebleached for ∼3 min (total across cells) prior to imaging to reduce the density of HALO-tagged molecules, background fluorescence, and the fluorescence interference from surrounding cells.

### Single molecule tracking: fast tracking (20 ms) analysis (PalmTracer)

Fast (20 ms) SMT raw image stacks were cropped in ImageJ prior to analysis to reduce their file size and therefore minimize analysis time. Fast SMT data was then analyzed using the PalmTracer plugin developed for Metamorph by the Sibarita group at the University of Bordeaux (30). As described by Bademosi and colleagues (31), here we used PalmTracer to localize and track molecules in order to obtain their trajectories, and to calculate the mean square displacement (MSD) and diffusion coefficient (*D*) for each trajectory. For molecule localization, we used a watershed of size 6. To reduce non-specific background, and to reduce the likelihood of mistracking, trajectories were filtered based on a minimum length of 8 and a maximum length of 1000, and a maximum travel distance of 5 μm. For visualization, a zoom of 8 with a fixed intensity and size of 1 was used. A spatial calibration of 100 nm and a temporal calibration of 20 ms was used. MSD fitting was performed using a log scale with a length of 4 and a step number of 1.

Color-coding of fluorescence intensity, diffusion coefficient and trajectory heatmaps was performed using ImageJ. Fluorescence intensity heatmaps range from black (no molecules detected) to white (highest density detected). Diffusion coefficient heatmaps range from white showing the highest diffusion coefficient to black showing the lowest diffusion coefficient. Trajectories are colored based on frame acquisition number.

Analysis files produced by PalmTracer were used as input for AutoAnalysis_SPT software (https://github.com/QBI-Software/AutoAnalysis_SPT/wiki) developed for the Meunier Laboratory at the Queensland Brain Institute by Dr Liz Cooper-Williams. AutoAnalysis_SPT software compiles the results obtained for each cell to obtain the average MSD, calculates the average area under the curve (AUC) of the MSD for each cell, generates a histogram showing the distribution of the different log_10_ diffusion coefficients, and calculates the mobile to immobile ratio for each cell. Here we used 10 MSD points, with a time interval of 0.02 s (20 ms acquisition time) and included trajectories with a minimum log_10_ diffusion coefficient of −5, and a maximum log_10_ diffusion coefficient of 1. For the log_10_ diffusion coefficient histogram, we chose a bin width of 0.1, and a mobile to immobile threshold of −1.5, determined mathematically using Equation (2):

(2) $$\begin{equation*}{\rm Log}{\left( D \right)_{{\rm threshold}}} = {\log_{10}}\left( {\frac{{{{0.100}^2}}}{{4\times4\times0.02}}} \right)\end{equation*}$$

where 0.100 is the pixel size in nm, 0.02 is the acquisition time in seconds and 4 refers to the number of MSD points used for fitting.

Individual MSDs for mobile and immobile fractions were calculated manually by segregating MSDs for each cell based on their log_10_ diffusion coefficient (less than −1.5 = immobile, greater than −1.5 = mobile), with trajectories with log_10_ diffusion coefficients higher than 1 and lower than −5 excluded. In GraphPad Prism (version 8.0) the AUC of the MSD was calculated for each cell using default settings.

**Figure 1.**
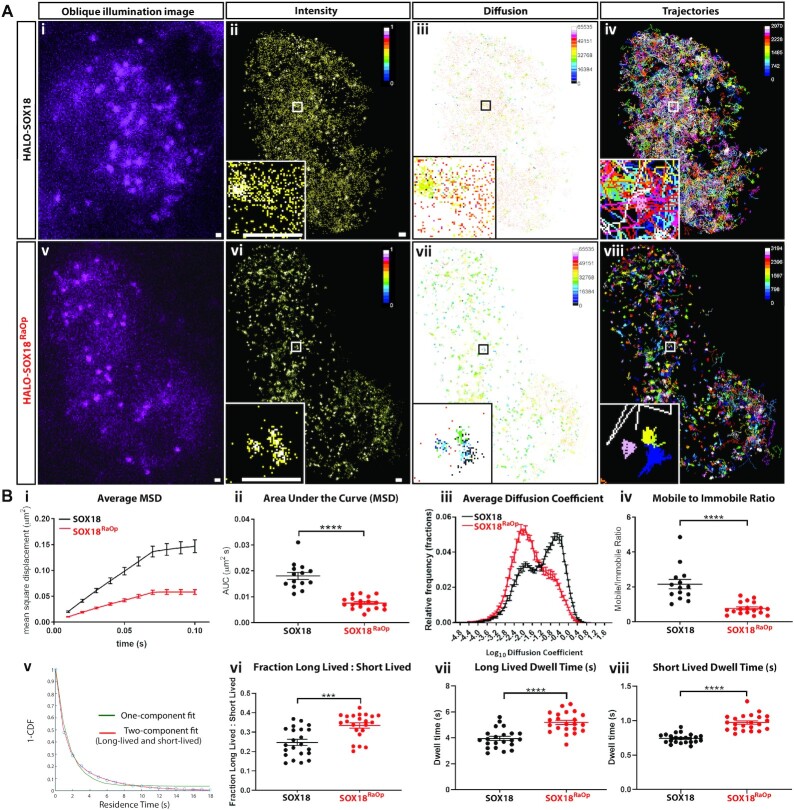
The SOX18^RaOp^ dominant-negative mutant protein displays impaired chromatin-binding dynamics. (**A**) (i and v) snapshot from oblique illumination live imaging. Heat maps: (ii and vi) fluorescence intensity (color code: white = highest intensity, black = lowest intensity), (iii and vii) diffusion coefficient (color code: warmer colors = higher mobilities, cooler colors = lower mobilities) and (iv and viii) trajectory maps (color code: based on trajectory frame). Scale bar = 0.5 μm. Example image number of trajectories: HALO-SOX18 = 2970, HALO-SOX18^RaOp^ = 3194. Average number of trajectories: HALO-SOX18 = 2453, HALO-SOX18^RaOp^ = 2215. (**B**) Quantification of the dynamics of HALO-SOX18 (black) and HALO-SOX18^RaOp^ (red). Top row: (i) the average mean square displacement (MSD; μm^2^), (ii) the area under the curve (AUC) of the average MSD for each cell (μm^2^s), (iii) the diffusion coefficient histogram for all cells (μm^2^s^−1^) and (iv) the mobile to immobile ratio for each cell. Threshold used to classify mobile and immobile molecules is Log_10_D = −1.5. Values for the mean ± s.e.m. are shown. *n* = 14 for HALO-SOX18 and *n* = 18 for HALO-SOX18^RaOp^ (*N* = 3). *t*-test (two-tailed, unpaired). **** *P* < 0.0001. Bottom row: (v) a two-component fit example for HALO-SOX18, (vi) fraction of long-lived to short-lived immobile events and dwell times of (vii) long-lived and (viii) short-lived immobile events (s). Values for the mean ± s.e.m. are shown. *n* = 22 for HALO-SOX18 and *n* = 22 for HALO-SOX18^RaOp^ (*N* = 3). Mann–Whitney *U*-test (two-tailed, unpaired). *** *P* < 0.001, **** *P* < 0.0001.

**Figure 2.**
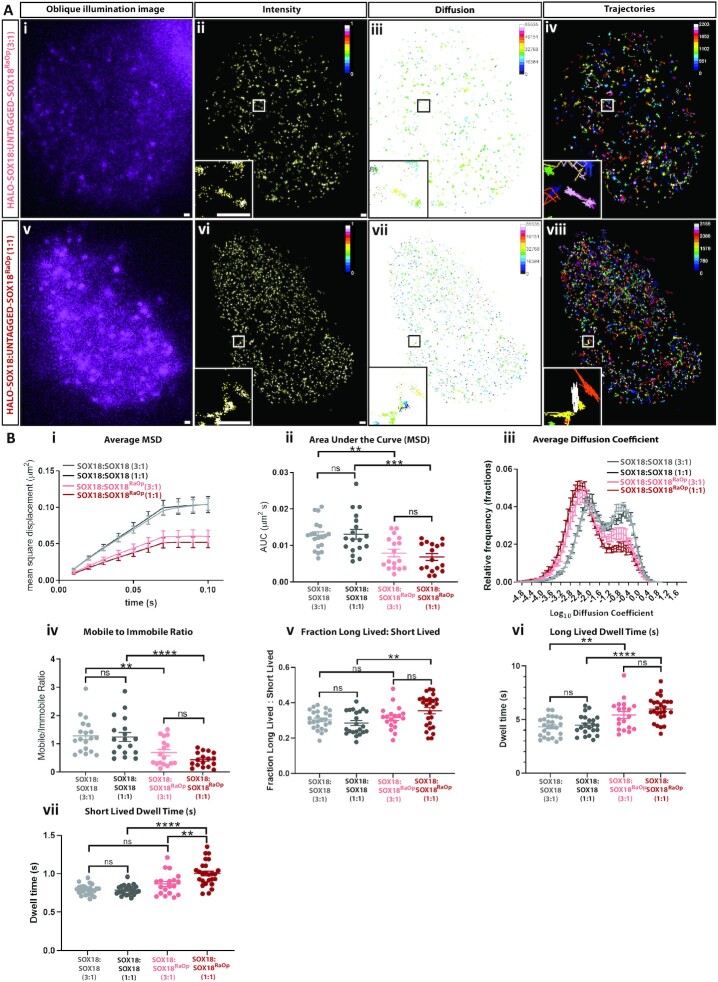
The SOX18^RaOp^ dominant-negative mutant protein directly interferes with the chromatin-binding dynamics of SOX18. (**A**) (i and v) snapshot from oblique illumination live imaging. Heat maps: (ii and vi) fluorescence intensity (color code: white = highest intensity, black = lowest intensity), (iii and vii) diffusion coefficient (color code: warmer colors = higher mobilities, cooler colors = lower mobilities) and (iv and viii) trajectory maps (color code: based on trajectory frame). Scale bar = 0.5 μm. Example image number of trajectories: HALO-SOX18:untagged-SOX18^RaOp^ (3:1) = 2203, HALO-SOX18:untagged-SOX18^RaOp^ (1:1) = 3155. Average number of trajectories: HALO-SOX18:untagged-SOX18 (3:1) = 2596, HALO-SOX18:untagged-SOX18 (1:1) = 1887, HALO-SOX18:untagged-SOX18^RaOp^ (3:1) = 1530, HALO-SOX18:untagged-SOX18^RaOp^ (1:1) = 1832. (**B**) Quantification of the dynamics of HALO-SOX18 with untagged SOX18 in a 3:1 ratio (light grey) or in a 1:1 ratio (dark grey) or with untagged SOX18^RaOp^ in a 3:1 ratio (light red) or 1:1 ratio (dark red). (i) average mean square displacement (MSD; μm^2^), (ii) area under the curve of the average MSD for each cell (μm^2^s), (iii) the diffusion coefficient histogram for all cells (μm^2^/s) and (iv) mobile to immobile ratio. Threshold to classify mobile and immobile molecules is log_10_*D* = −1.5. Values for the mean ± s.e.m. are shown. *n* = 19, *n* = 18, *n* = 17 and *n* = 17 (*N* = 3). Data was log transformed for ANOVA analysis, raw data is displayed. Statistical significance was determined by a Tukey post-hoc test. ** *P* < 0.01, *** *P* < 0.001, **** *P* < 0.0001, ns = non-significant (*P* > 0.05). (v) Fraction of long-lived to short-lived immobile events, and dwell times of (vi) long-lived and (vii) short-lived immobile events (s). Values for the mean ± s.e.m. are shown. *n* = 25, *n* = 22, *n* = 19 and *n* = 26 (*N* = 3). Short-lived dwell time data was reciprocal (1/Y) transformed for ANOVA analysis, raw data is displayed. Statistical significance was determined by a Tukey post-hoc test. ** *P* < 0.01, **** *P* < 0.0001, ns = non-significant (*P* > 0.05).

Values for the mean ± s.e.m. were plotted into GraphPad Prism. Cells with significant drift or less than 1000 trajectories were excluded from analysis, with the exception of HALO-SOX18^DIM^ (and the HALO-SOX18 condition obtained on the same day for comparison) for which cells with less than 800 trajectories were excluded. A robust regression and outlier removal (ROUT) outlier test using default settings (*Q* = 1%) in GraphPad Prism was performed to identify and remove outliers. Significance of the AUC of the MSD, and the mobile to immobile ratio of the diffusion coefficient histogram was assessed. For data with two categories unpaired two-tailed t-tests were performed (Figure 1B ii and iv, Supplementary Figures S3A, B and S4 ii and iv, S11J ii and iv). For data with more than two categories, ANOVAs were performed. Data was left untransformed if the assumptions of homoscedasticity and normality of residuals required for ANOVA was already met (Supplementary Figures S7 ii and iv, S12 ii), or log transformed in order to meet these assumptions (Figures 2B ii and iv, 4C iii and v, and Supplementary Figure S12 iv). Pairwise comparisons were performed using a Tukey post-hoc test.

### Single molecule tracking: slow tracking (500 ms) analysis

Slow (500 ms) SMT raw image stacks were cropped in ImageJ prior to analysis to reduce their file size and therefore minimize analysis time.

Slow tracking SMT data was analysed via a MATLAB pipeline using MATLAB version R2015a as previously published by Chen and colleagues (2). Here, we used this MATLAB pipeline to assess whether the immobile fraction consists of one or two types of dwell times (long-lived, a few seconds and short-lived, less than 1 s), calculate the fraction of long-lived to short lived dwell times and the length of time for which they occurred. This pipeline first uses the MATLAB script SLIMfast.m to localise molecules, which is a modified version of the multiple-target tracing (MTT) algorithm reported by Sergé and colleagues (32). SLIMfast.m is available on the eLife website (https://doi.org/10.7554/eLife.22280.022). SLIMfast batch processing was performed using an error rate of 10^–7^, a detection box of 7 pixels, maximum number of iterations of 50, a termination tolerance of 10^–2^, a maximum position refinement of 1.5 pixels, an N.A. of 1.46, a PSF scaling factor of 1.35, and 20.2 counts per photon, an emission of 590 nm, a lag time of 500 ms and a pixel size 100 nm. Trajectory creation was performed using the maximum expected diffusion coefficient of 0.1 μm^2^/s. Following this, the MATLAB script Calculatelength_2fitting_v3.m (available on request: liuz11@janelia.hhmi.org) is used to calculate the lifetime of molecules for each cell, and fits one and two-component exponential decay curves to this data using Equation (3):

(3) $$\begin{equation*}F\left( t \right) = {f_l}{e^{ - t/{\tau _s}}} + \left( {1 - {f_i}} \right){e^{ - t/{\tau _{ns}}}}\end{equation*}$$

The two-component model derives the average dwell time for specific and non-specific fractions and the ratio between these. Values extracted from this two-component model were plotted into GraphPad Prism, with the mean ± s.e.m. shown. Cells with significant drift were excluded from analysis. A robust regression and outlier removal (ROUT) outlier test using default settings (*Q* = 1%) in GraphPad Prism was performed to identify and remove outliers. Significance of the long-lived to short-lived fraction, long-lived dwell time and short-lived dwell time was assessed. For data with two categories a Mann–Whitney *U*-test was performed (Figure 1B vi-viii, Supplementary Figures S4 v–vii, S11J v–vii). For data with more than two categories ANOVAs were performed. Data was left untransformed if the assumptions of homoscedasticity and normality of residuals required for ANOVA was already met (Figures 2B v and vi, 4C vii, 5I, J, Supplementary Figures S7 v–vii and S12 v), log transformed in order to meet these assumptions (Figure 4C viii), or reciprocal (1/*Y*) transformed in order to meet these assumptions (Figures 2Bvii, 4C vi). Pairwise comparisons were performed using a Tukey post-hoc test.

### Single molecule tracking: fast tracking (20 ms) analysis (SLIMfast)

The same cropped images used for PalmTracer analysis were also used as input for analysis in SLIMfast, in order to compare trends across different track-based analysis pipelines. SLIMfast batch processing was performed using an error rate of 10^–6^, a detection box of 7 pixels, maximum number of iterations of 50, a termination tolerance of 10^–2^, a maximum position refinement of 1.5 pixels, an N.A. of 1.46, a PSF scaling factor of 1.35, and 20.2 counts per photon, an emission of 590 nm, a lag time of 20 ms, and a pixel size of 100 nm. Trajectory creation was performed using the max expected diffusion coefficient of 3 μm^2^/s. Trajectories with less than 8 tracks were excluded. Following this, custom MATLAB scripts were used to select individual ROIs and produce diffusion coefficient histograms and trajectory maps as well as a combined diffusion coefficient histogram of all ROIs analyzed. The log_10_ diffusion coefficients obtained from MATLAB were plotted into GraphPad Prism in order to compare the log_10_ diffusion coefficient histogram to the one generated using PalmTracer.

### Single molecule tracking: fast tracking (20 ms) analysis (Spot-On)

In order to compare the diffusion coefficient and bound fraction trends produced by track-based methods to those estimated by a jump-distanced based algorithm, we also analysed the data using the MATLAB package Spot-On, developed by Hansen and colleagues (33). PalmTracer and SLIMfast output files were converted to Spot-On input files using custom MATLAB scripts. Default Spot-On settings were used, aside from localization error from data marked as yes, and performing two separate analyses with NumberOfStates set to 2 for 2-component analysis, and NumberOfStates set to 3 for 3-component analysis. Values were plotted into GraphPad Prism with the mean ± s.e.m. shown. Statistical significance of pairwise comparisons were assessed using a Mann–Whitney *U*-test (Supplementary Figures S5C and D).

### Cell preparation for ChIP-seq

HeLa cells were seeded in 3 × 15 cm dishes per condition at 2.65 × 10^6^ cells/dish, and then transfected for 24 h with the following combination of plasmids: (i) 15 μg pcDNA3.1 glomyc-SOX18 and 15 μg untagged-SOX18; (ii) 15 μg pcDNA myc-SOX18^RaOp^ and 15 μg untagged-SOX18^RaOp^; (iii) 15 μg pcDNA3.1 glomyc-SOX18 and 15 μg untagged-SOX18^RaOp^. Transfections were performed using the X-tremeGENE 9 Transfection Reagent kit (Roche) as per manufacturer's instructions, with a DNA to X-tremeGENE 9 ratio of 1 μg to 3 μl.

Cells were then fixed by adding 2 ml of molecular biology grade formaldehyde solution (11% formaldehyde, 0.1 M NaCl, 1 mM EDTA, 50 mM HEPES) in the cells' existing media (20 ml) for 15 min at room temperature with agitation. Fixing was quenched by adding 1.155 ml of 2.5 M glycine solution for 5 min at room temperature. Cells were then scraped and pelleted at 800 g for 10 min at 4°C. Pellets were washed with 10 ml ice-cold PBS, pooled, and then centrifuged at 800 g for 10 min at 4°C. Resuspension of pellets was performed in 10 ml ice-cold PBS supplemented with cOmplete protease inhibitor (Roche), and then centrifuged at 800 g for 10 min at 4°C. Supernatant were removed completely, and the resultant pellets were snap-frozen in dry ice, and then stored at −80°C until analysis.

### Chromatin Immunoprecipitation

Samples were sent to Active Motif (Carlsbad, CA) for ChIP-Seq. Active Motif prepared chromatin, performed ChIP reactions, generated libraries, sequenced the libraries and performed basic data analysis. Chromatin was isolated by adding lysis buffer, followed by disruption with a Dounce homogenizer. Lysates were sonicated and the DNA sheared to an average length of 300–500 bp with Active Motif's EpiShear probe sonicator (cat# 53051). Genomic DNA (Input) was prepared by treating aliquots of chromatin with RNase, proteinase K and heat for de-crosslinking, followed by SPRI beads clean up (Beckman Coulter) and quantitation by Clariostar (BMG Labtech). Extrapolation to the original chromatin volume allowed determination of the total chromatin yield.

An aliquot of chromatin (40 μg) was precleared with protein G agarose beads (Invitrogen). Genomic DNA regions of interest were isolated using 6 μg of antibody against Myc-tag (Abcam, ab9132, Lot# GR3325755-1). Complexes were eluted from the beads with SDS buffer, and subjected to RNase and proteinase K treatment. Crosslinks were reversed by incubation overnight at 65°C, and ChIP DNA was purified by phenol-chloroform extraction and ethanol precipitation.

### ChIP sequencing (Illumina)

Illumina sequencing libraries were prepared from the ChIP and Input DNAs by the standard consecutive enzymatic steps of end-polishing, dA-addition, and adaptor ligation. Steps were performed on an automated system (Apollo 342, Wafergen Biosystems/Takara). After a final PCR amplification step, the resulting DNA libraries were quantified and sequenced on Illumina's NextSeq 500 (75 nt reads, single end). Reads were aligned to the human genome (hg38) using the BWA algorithm (default settings). Duplicate reads were removed and only uniquely mapped reads (mapping quality ≥ 25) were used for further analysis. Alignments were extended in silico at their 3′-ends to a length of 200 bp, which is the average genomic fragment length in the size-selected library, and assigned to 32-nt bins along the genome. The resulting histograms (genomic ‘signal maps’) were stored in bigWig files. Peak locations were determined using the MACS algorithm (v2.1.0) with a cut-off of *P*-value = 1e−7. Peaks that were on the ENCODE blacklist of known false ChIP-Seq peaks were removed. Signal maps and peak locations were used as input data to Active Motifs proprietary analysis program, which creates Excel tables containing detailed information on sample comparison, peak metrics, peak locations and gene annotations.

### AlphaScreen assay

Here, we screened for interactions between SOX18 protein partners and SOX18 recessive mutants (W95R and A104P) in pairs, as well as interactions between SOX18 protein partners and SOX18 dominant-negative mutants (Q161*, E169*, G204* and C240*) in pairs. The SOX18 protein partners used in this screen were identified previously in Overman *et al.* (34) using the Rapid Immunoprecipitation Mass spectrometry of Endogenous proteins (RIME) method developed by Mohammed *et al.* (35), and were validated using AlphaScreen (36).

AlphaScreen assays were performed to assess interactions between pairs of proteins (protein A and protein B) as previously described (36). Buffer A (25 mM HEPES, 50 mM NaCl) and Buffer B (25 mM HEPES, 50 mM NaCl 0.001% NP40, 0.001% casein) were prepared. 30 nM of protein A plasmid DNA and 60 nM of protein B plasmid DNA were added to 10 μl of *Leishmania tarentolae* cell-free lysate (LTE lysate) and the mixture was incubated at 27°C for 3.5 h to induce co-expression of the two proteins. The co-expressed protein/LTE mixture was serially diluted (1:10) in Buffer A. 12.5 μl (0.4 μg) of Anti-cMyc coated Acceptor beads in Buffer B, 2 μl of the co-expressed proteins in LTE lysate/Buffer A, and 2 μl of GFP-nanotrap conjugated to biotin, diluted in Buffer A, were added to each well of a Proxiplate-384 Plus plate (PerkinElmer). Plates were incubated at room temperature for 45 min, then 2 μl (0.4 μg) of streptavidin-coated donor beads in Buffer A were added, followed by further incubation at room temperature for 45 min in the absence of light. To collect the signal, an Envision Multilabel Plate Reader (PerkinElmer) was used with an excitation of 680/30 nm for 180 ms and emission of 570/100 nm after 37 ms, as per manufacturer's recommendations. Experiments were performed as technical duplicates for each biological triplicate. The Binding Index (BI) was calculated using Equation (4):

(4) $$\begin{equation*}{\rm BI} = \left[ {\frac{{\left( {I - {I_{{\rm neg}}}} \right)}}{{\left( {{I_{{\rm ref}}} - {I_{{\rm neg}}}} \right)}}} \right]\times100\end{equation*}$$

where *I* is the maximal intensity for each protein pair, *I*_neg_ is the lowest intensity for each protein pair (background) and *I*_ref_ is a reference pair (SOX18−SOX18). Data was fit to a 3-parameter non-linear regression curve using GraphPad Prism.

Of note, all full-length transcription factors tested in the SOX18 interactome were produced using a cell-free lysate system. Based on this, some proteins were codon optimized in order to provide a production yield that is compatible with the AlphaScreen assay. Furthermore, here we used naturally occurring recessive (W95R and A104P) and dominant-negative (Q161* and C240*) mutations that have previously been reported in Human (6,7,37,38), as well as two engineered dominant-negative mutants that mimic naturally occurring mutations in Human (E169* which mimics E169Gfs*14 reported in Human, and G204* which is not reported in humans but mimics the premature truncation of the transcriptional activation domain) (39,40).

### JF549 dye titration

A titration of JF549 dye was performed in order to identify an ideal dye concentration that saturates HALO-SOX18 for use in N&B and cRICS experiments (Supplementary Figure S8). HeLa cells were seeded and incubated (24 h at 37°C), prior to transfection with 1 μg of plasmid DNA as per manufacturer's instructions (Lipofectamine 3000) and incubated further (24 h at 37°C). Cells were incubated with different JF549 dye concentrations (2, 10, 100 and 1000 nM) for 15 min prior to confocal imaging. The intensity of each dye concentration was then quantified. Data was log transformed for ANOVA analysis, and statistical significance was determined by a Tukey post-hoc test. A non-significant difference between neighboring concentrations was used to identify the concentration at which HALO-SOX18 becomes saturated with JF549 dye.

### Confocal microscopy for fluorescence fluctuation spectroscopy

All microscopy measurements were performed on an Olympus FV3000 laser scanning microscope coupled to an ISS A320 Fast FLIM box for fluorescence fluctuation data acquisition. A 60× water immersion objective 1.2 NA was used for all experiments and live HeLa cells were imaged at 37°C in 5% CO_2_. For single color fluorescence fluctuation spectroscopy experiments (e.g. N&B) SOX7 (Supplementary Figure S9A and D), SOX18 (Figure 5C and D, and Supplementary Figure S9E), SOX18 DNA-binding and homodimerization mutants (SOX18^AH1^ and SOX18^DIM^; Supplementary Figure S11A–D) and SOX18 dominant-negative mutant SOX18^RaOp^ (Figure 5C and D) were labeled 15 min prior to imaging via direct addition of 1 μM of JF549 Halo-tag dye, where JF549 was excited by a solid-state laser diode operating at 561 nm. The fluorescence signal was then directed through a 405/488/561 dichroic mirror to remove laser light and the JF549 emission collected through a 550 nm long pass filter by an external photomultiplier detector (H7422P-40 of Hamamatsu) fitted with a 620/50 nm bandwidth filter. A 100-frame scan acquisition of the JF549 signal was then collected by selecting a region of interest within a HeLa cell nucleus at zoom 20, which for a 256 × 256-pixel frame size resulted in a pixel size of 41 nm. The pixel dwell time was set to 12.5 μs, which resulted in a line time of 4.313 ms and a frame time of 1.108 s.

For the two-color experiments (e.g. cRICS) (Figure 5E-H, Supplementary Figures S9B and C, and S11E–I) SOX7, SOX18, SOX18^AH1^, SOX18^DIM^ and SOX18^RaOp^ were co-labelled with 500 nM of JF549 as well as 500 nM of JF646 and these two dyes were excited by solid-state laser diodes operating at 561 nm and 640 nm (respectively). For the two-color experiment interrogating SNAP-SOX18/HALO-SOX18^RaOp^ heterodimers, 500 nM of snap646 and 500 nM of JF549 was used (Figure 5G and H). The fluorescence signal was then directed through a 405/488/561/640 dichroic mirror to remove laser light and the JF549 versus JF646 emission was detected by two internal GaAsp photomultiplier detectors set to between the following bandwidths: JF549 570–620 nm, JF646 650–750 nm. A two channel 100-frame scan acquisition of the JF549 and JF646 signal was then collected simultaneously employing the same settings described above for the single channel experiment.

### Number and brightness (N&B)

Number and brightness (N&B) analysis of single-channel frame scan acquisition was performed using a moment-based analysis as described in previously published papers (41–43). Briefly, in each pixel of the frame scan there is an intensity fluctuation that has an average intensity (first moment) and a variance (second moment). The ratio of these two properties describes the apparent brightness (*B*) of the molecules that give rise to the intensity fluctuation. In the case of a photon counting detector, the true molecular brightness (*ϵ*) of the molecules is related to the measured apparent brightness (*B*) by Equation (5):

(5) $$\begin{equation*}B = \varepsilon + 1\end{equation*}$$

where 1 is the brightness contribution from the photon-counting detector (41). Calibration of the monomeric brightness of JF549, via measurement of monomeric SOX7 tagged with this fluorophore (Supplementary Figure S9A and D), enabled extrapolation of JF549 tagged SOX18 dimers and oligomers (Supplementary Figure S9E), as well as quantitation of the fraction of pixels within a given N&B acquisition that contain each of these species (e.g. fraction of dimer in Figure 5D). Artefacts due to cell movement or cell bleaching were subtracted from acquired intensity fluctuations via use of a moving average algorithm (*N* = 10 frames). All brightness calculations were carried out from the NB page in SimFCS from the Laboratory for Fluorescence Dynamics (www.lfd.uci.edu). A robust regression and outlier removal (ROUT) outlier test using default settings (*Q* = 1%) in GraphPad Prism was performed to identify and remove outliers. Statistical significance was assessed for data with two categories using a Mann–Whitney *U*-test (Figure 5D), whereas data with more than two categories was assessed by ANOVA and was log transformed in order to meet the assumptions of homoscedasticity and normality of residuals (Supplementary Figure S11D) required for ANOVA analysis. Pairwise comparisons were performed using a Tukey post-hoc test.

### Raster image correlation spectroscopy (RICS) and cross-RICS (cRICS)

Raster image correlation spectroscopy (RICS) and cross-RICS (cRICS) analysis of two-channel frame scan acquisitions was performed via use of the RICS and cRICS spatiotemporal correlation functions described in previously published papers (44–46). Briefly, the fluorescence intensity recorded in each frame (*N* = 100) within each channel (CH1 and CH2) was spatially correlated via application of the RICS function, and then spatially cross-correlated between channels (CC) via application of the cRICS function, alongside a moving average algorithm (*N* = 10 frames). In the case of SOX18, the three-dimensional (3D) RICS correlation profile in CH1 and CH2 fit to a 2-component 3D diffusion model (Supplementary Figure S11H), while the 3D cRICS cross-correlation profile CC fit a 1-component 3D diffusion model (Supplementary Figure S11I). In each case, the recovered amplitudes (*G*) as well as diffusion coefficients (*D*) were recorded to enable the fraction of SOX18 molecules in a hetero-dimeric or homo-dimeric complex to be calculated, and in either case, the quantification of the mobility of the complex. A robust regression and outlier removal (ROUT) outlier test using default settings (*Q* = 1%) in GraphPad Prism was performed to identify and remove outliers. Statistical significance for data with two categories was assessed using a Mann–Whitney *U*-test (Figure 5F), data with more than two categories not involving comparison to an internal reference control were assessed using a Kruskal–Wallis test (Figure 5G and Supplementary Figure S11I), and data with more than two categories involving comparison to an internal reference control (e.g. SOX7 is monomeric and does not form homodimers) were assessed using a one-tailed t-test with a mu of 0 (expected SOX7 homodimer % = 0) (Figures 4Ci and 5H).

### Statistical analysis

All statistical analyses for SMT, N&B and cRICS were performed in GraphPad Prism (version 8.0). A robust regression and outlier removal (ROUT) outlier test using default settings (*Q* = 1%) in GraphPad Prism was performed to identify and remove outliers. Data with two categories: significance was assessed for fast SMT using unpaired two-tailed t-tests, and for slow SMT, N&B and cRICS analyses using two-tailed unpaired Mann-Whitney U-tests. Data with more than two categories: SMT, N&B and cRICS data was either (i) left untransformed if it already met the assumptions of homoscedasticity and normality of residuals required for ANOVA analysis and pairwise comparisons were performed using a Tukey test, (ii) transformed using either log transformation or reciprocal (1/*Y*) transformation to meet these assumptions then tested by ANOVA and pairwise comparisons using a Tukey post-hoc test to assess significance, (iii) assessed for significance using a Kruskal–Wallis test if the assumptions required for ANOVA could not be met or (iv) assessed for significance using a one-tailed *t*-test with a mu of 0 if comparison to a dimerization negative control was used. ns = non-significant (*P* > 0.05), * <0.05, ** <0.01, *** <0.001 and **** < 0.0001.

### Histone and RNA-polymerase II SOX18 ChIP-seq overlap analysis

Previously reported SOX18-myc ChIP-seq datasets obtained in HUVECs by Overman *et al.* (34) (https://www.ebi.ac.uk/arrayexpress/experiments/E-MTAB-4481/) was used to compare the overlap of total-SOX18 and SOX18 homodimer only (36) ChIP-seq peaks, with active and repressive histone mark and RNA polymerase II ChIP-seq peaks in HUVECs which were made publicly available for use by the ENCODE consortium (https://www.ebi.ac.uk/arrayexpress/experiments/E-GEOD-29611/). The online webtool EpiExplorer was used to assess the percentage of overlap (47). Here, we defined overlap as an overlap of at least 50% of the base pairs at the two closest edges of the SOX18 peak and the respective histone/RNA polymerase II peak.

### Fluorescence fluctuation spectroscopy aggregation assay

Fluorescence fluctuation spectroscopy aggregation assays were performed as previously described (48). Each GFP-tagged protein was expressed in LTE by adding 30 nM plasmid DNA to the lysate and incubating at 27°C for 2.5 h. Following incubation, the sample was subdivided into twelve 5 μl samples, which were subjected to a temperature gradient from 30°C to 60°C (Eppendorf Mastercycler Gradient PCR machine). Samples were then diluted 11 times and placed on a custom single molecule setup for analysis. Five 30 second fluorescence time traces were obtained for each sample. The Brightness (*B*) of each trace was calculated using Equation (6):

(6) $$\begin{equation*}B = \frac{{{{\rm SD}^2}}}{\mu }\end{equation*}$$

where SD is the standard deviation, and μ the mean of the data (49). The data were averaged between the different repeats and replicates. The increase in brightness reflects the increased presence of aggregates due to thermal denaturation. To evaluate the conserved ability to bind DNA, the aggregation assays were repeated in the presence of 1 μM of SOX18 single consensus sequence (36). Binding to the consensus sequence results in an increase in thermal stability and a shift of the curve towards higher temperatures.

## RESULTS

In order to analyse the search pattern mechanism of SOX18 and its dominant-negative mutant counterpart SOX18^RaOp^, we took advantage of self-labeling Halo-tag technology (50,51) and used it in combination with three single-molecule resolution imaging techniques - single molecule tracking (SMT) (1,2,52–56) to measure the overall chromatin-binding dynamics, number and brightness (N&B) (41–43,57) to obtain the oligomeric distribution and cross-raster image correlation spectroscopy (cRICS) (44–46) using two spectrally distinct Halo-tag dyes (JF549 and JF646) to validate homodimer formation and to obtain their ensemble mobility. An overview describing the steps involved in, and biological information obtained from using each of these imaging techniques, is described in Supplementary Figure S1.

Of note, SOX18 is a key regulator of blood vessel development, and as a result is highly expressed in blood vascular endothelial cells. In support of this, CAGE data from the FANTOM5 consortium shows that SOX18 is the most enriched TF in Human Umbilical Vascular Endothelial Cells (HUVECs) (https://fantom.gsc.riken.jp/cat/v1/#/genes/ENSG00000203883.5). Here, we intend to assess the mode of action of a dominant-negative SOX18 mutant (SOX18^RaOp^) by modulating the levels of the wild-type SOX18 protein. For this reason, we have performed SMT, N&B and cRICS experiments in HeLa cells which have no detectable endogenous expression of SOX18 (Supplementary Figure S2A).

### The SOX18^RaOp^ dominant-negative mutant form is a potent transcriptional repressor

Here, we set out to investigate the molecular mode of action of disease-causing mutations that give rise to a dominant-negative SOX18 protein. Such a truncated protein is still able to bind to DNA but fails to activate gene transcription (39). The Ragged mouse model exhibits natural mutations in the SOX18 gene—an allelic series of mutations associated with a broad range of phenotypic outcomes (from mild to severe vascular, renal and hair follicle defects) (23). The most severe Ragged mutant is known as opossum (SOX18^RaOp^), characterized by a point deletion within the C-terminal transactivation domain causing a frameshift that scrambles the rest of the transactivation domain before resulting in a premature stop codon (Supplementary Figure S2B). Due to the nature of its mutation, SOX18^RaOp^ is truncated and forms a shorter version of SOX18. To ensure that this size difference does not significantly affect the protein level by altering the degradation rate, we assessed the concentration of HALO-SOX18 and HALO-SOX18^RaOp^ at the whole cell and nuclear level (Supplementary Figure S2C). By comparing whole cell lysates, we found that HALO-SOX18 and HALO-SOX18^RaOp^ are expressed at comparable levels, and by comparing nuclear extracts we found that more HALO-SOX18^RaOp^ accumulates in the nucleus. This shows that in addition to allele overexpression reducing protein functionality to <25% as described by Veitia (5), some dominant-negative mutations increase the intranuclear concentration of a TF, therefore amplifying its deleterious potential.

To explore the molecular mode of action of SOX18^RaOp^ during gene transactivation, we performed a luciferase assay using a synthetic VCAM-1 promoter fragment as a readout for SOX18 transcriptional activity (29) (Supplementary Figure S2D). HALO-SOX18 efficiently activated VCAM-1 promoter activity, whereas SOX18^RaOp^ failed to do so. Further, SOX18^RaOp^ prevented HALO-SOX18 from transactivating the VCAM-1 promoter fragment. These results validate that the addition of the HALO-tag does not compromise SOX18 transcriptional activity, nor does it prevent the dominant-negative mode of action of SOX18^RaOp^. The addition of an excess of HALO-SOX18 construct in a dose-dependent manner to outcompete the repressive effect of SOX18^RaOp^ failed to rescue the lack of transcriptional activity caused by the mutant protein. Strikingly, even at a ratio of 30:1 HALO-SOX18 to SOX18^RaOp^, the transcriptional activity was not restored, suggesting that gene dose response of the wild-type allele is not sufficient to compensate the dominant-negative mechanism of the mutant protein. This is in accordance with a dominant-negative phenotype, and what has been previously mathematically modelled by Veitia (5).

Previous studies have used single molecule tracking (SMT) to uncover functional aspects of the search patterns of TFs, and by doing so have shown that changes in their chromatin-binding behavior reflects changes in their gene target selection and activity (1,2,53–56). Here, we hypothesized that altered chromatin-binding dynamics may form an important part of the dominant-negative mode of action of SOX18^RaOp^. To explore this, we generated a HALO-SOX18^RaOp^ construct using HALO-SOX18 as a backbone, transiently transfected HeLa cells with either HALO-SOX18 or HALO-SOX18^RaOp^, and performed fast (20 ms acquisition, 6000 frames) and slow (500 ms acquisition, 500 frames) SMT (Figure 1A and B, Videos S1 and S2). These two different acquisition speeds provide information on the trajectories of the unbound diffusing and immobile chromatin-bound states (fast acquisition), and the different types of dwell times on the chromatin (slow acquisition).

Focusing on the chromatin-binding dynamics in the wild-type scenario, we found that an average of 34% of SOX18 molecules are immobile, with the rest being mobile and diffusing (Figure 1B). Of this immobile fraction, by comparing one and two-component fit models we identify that there are at least two types of immobile populations with different dwell times, which is in accordance with what has been reported previously for SOX2 (2) and other TFs (2,53–55,58). Here, we found that SOX18 had an average long-lived dwell time of 3.94 s and an average short-lived dwell time of 0.74 s (Figure 1B), in accordance with previously reported long-lived dwell times that typically last a few seconds (∼5–14.6 s) and short-lived dwell times that are typically 1 s or less (∼0.03–1.85 s) (2,53,54,58). Additionally, we found that long-lived binding events accounted for one quarter of SOX18 immobile events. Previous SMT studies have demonstrated that these short-lived and long-lived dwell times are due to interactions with non-specific random and specific target chromatin sites respectively, notably via the use of DNA-binding and homodimerization mutants (2,54).

When comparing the chromatin-binding dynamics of SOX18 to SOX18^RaOp^, we observed a significant difference in the search pattern of HALO-SOX18 and HALO-SOX18^RaOp^ already at the level of the raw data used for fast SMT analysis (Figure 1A). An example of this is highlighted in Video S1. HALO-SOX18^RaOp^ appears to be a lot less mobile than its wild-type counterpart, with what appears to be more immobile chromatin binding events, which remain immobile for longer (Video S1, Figure 1A). The intensity and diffusion coefficient heatmaps show two main types of TF behaviors – immobile chromatin-binding events represented by distinct higher intensity regions associated with lower diffusion coefficients, and scattered between these, mobile diffusion events represented by lower intensity regions associated with higher diffusion coefficients (Figure 1A). Based on this readout, SOX18^RaOp^ shows an overall lower mobility than SOX18, with more immobile chromatin-binding events and less diffusional events. In support of this, the trajectory maps show that SOX18^RaOp^ appears to have more trajectories contained within small areas suggesting greater immobility. By contrast, SOX18 appears to have more trajectories that explore less restricted areas, suggesting a higher diffusive behavior.

By quantifying the trajectories of HALO-SOX18 and HALO-SOX18^RaOp^ obtained via fast SMT (Figure 1B), we observed that overall SOX18^RaOp^ has a significantly lower mobility. This is based on its lower average mean square displacement (MSD), and its significantly higher immobile fraction represented by a higher peak in the diffusion coefficient histogram. The average MSDs for all trajectory types (mobile and immobile) shown in Figure 1B were separated into average MSDs for immobile (Supplementary Figure S3A) and mobile trajectories (Supplementary Figure S3B). Comparing the average MSDs for immobile and mobile trajectories for HALO-SOX18 and HALO-SOX18^RaOp^ shows that while the mobility of HALO-SOX18^RaOp^ decreases in both immobile and mobile fractions, the mobile diffusing fraction is affected the most. Diffusion coefficient histograms for all cells are shown in Supplementary Figure S3C and D. Despite heterogeneity in the diffusion coefficient histograms across cells, a clear trend can be observed where HALO-SOX18^RaOp^ shifts towards the immobile fraction.

By comparing the fraction of long-lived to short-lived immobile events obtained by slow tracking, and how long they occurred for, we found that SOX18^RaOp^ had a higher fraction of long-lived immobile events, and both long-lived and short-lived immobile events were longer for SOX18^RaOp^ than SOX18 (Figure 1B). To assess whether a change in chromatin-binding stability may play a role in the difference in behavior observed for SOX18^RaOp^, we deleted the first alpha helix (AH1) of the HMG DNA-binding domain (HALO-SOX18^AH1^; Supplementary Figure S4). This mutant is still capable of binding to DNA via alpha helix 2 (AH2) but with lower affinity. As anticipated, decreasing the DNA-binding stability of SOX18 produced the opposite behavior observed for HALO-SOX18^RaOp^, with SOX18^AH1^ displaying an increased MSD, lower chromatin-bound and long-lived binding fractions, and shorter dwell times. Of note, when SMT analysis was performed, only cells expressing a sufficient amount of HALO-SOX18^AH1^ in the nucleus were included, with cells that had less than 1000 trajectories excluded. Taken together these results indicate that one of the hallmarks of the non-functional SOX18^RaOp^ TF is a significant increase in chromatin-binding stability, which may also explain the enhanced nuclear concentration observed for SOX18^RaOp^ in Supplementary Figure S2C.

Here, we used the Metamorph plugin PalmTracer to obtain average diffusion coefficients for the bound and unbound fractions of SOX18 (0.02 μm^2^/s and 0.43 μm^2^/s) and SOX18^RaOp^ (0.02 and 0.27 μm^2^/s). Furthermore, we used PalmTracer to estimate the bound fraction of SOX18 (34%) and SOX18^RaOp^ (58%). Previous studies using other algorithms to investigate transcription factor mobility have estimated higher diffusion coefficients of ∼2–10 μm^2^/s (2,55). An example of this is SOXB member SOX2 (bound = 0.15 μm^2^/s, unbound = 2.1 μm^2^/s) (2). We investigated this by comparing the estimates given by different SMT analyses (PalmTracer, SLIMfast and Spot-On) using the same datasets, and found that the major cause for this discrepancy is due to the algorithm used which is discussed in length in the supplementary data (Supplementary Figure S5, Supplementary Table S2). Importantly, the trends observed for SOX18 and SOX18^RaOp^ using PalmTracer remained the same regardless of the algorithm used.

### The SOX18^RaOp^ mutant protein derails the chromatin-binding dynamics of SOX18

The dominant-negative form of SOX18 is embryonic lethal when homozygous, leaving only heterozygous individuals to survive this condition (21,23,28). This implies that in the case of bi-allelic expression in a heterozygous scenario both SOX18 and SOX18^RaOp^ co-exist in the same cells at the same time. In order to assess the direct interference of SOX18^RaOp^ on SOX18 activity we next set out to measure the chromatin-binding dynamics of the wild-type protein in presence of the mutant protein.

To achieve this, SMT analysis was performed using transiently co-transfected HeLa cells with HALO-SOX18 in a 3:1 or 1:1 ratio with either untagged-SOX18^RaOp^, or untagged-SOX18 as a protein expression control (Figure 2A and B). Example SMT videos comparing each of these conditions can be found in Videos S3 and S4. By quantifying and comparing the trajectories for HALO-SOX18:SOX18 (3:1) and HALO-SOX18:SOX18 (1:1), we found that there were no significant differences in the chromatin-binding dynamics of HALO-SOX18 despite increasing the amount of untagged protein. By using HALO-SOX18:SOX18 (3:1) and (1:1) as a point of comparison to HALO-SOX18:SOX18^RaOp^ (3:1) and (1:1) respectively, we found a significant difference in the behavior of HALO-SOX18 upon the addition of untagged-SOX18^RaOp^. By looking at the intensity, diffusion coefficient and trajectory maps, we found that HALO-SOX18 upon the addition of untagged-SOX18^RaOp^ behaves in a similar fashion to what was previously observed for HALO-SOX18^RaOp^, with HALO-SOX18 trajectories now having more distinct high intensity foci associated with lower diffusion coefficients and exploring less area (Figure 2A). Examples of new behaviors are shown within the insets. These differences in behavior indicate that SOX18^RaOp^ greatly alters the search pattern of SOX18.

Quantification of HALO-SOX18 trajectories in the presence of SOX18^RaOp^ revealed that the addition of the dominant-negative protein decreased the overall mobility of SOX18 (Figure 2B). Further, it also increased the immobilized fraction of the total SOX18 population, increased the fraction of immobile events that occurred for a long period of time, and extended the length of time of both long-lived and short-lived immobile events. Based on these observations, SOX18^RaOp^ appears to poison the wild-type TF with a SOX18^RaOp^-like molecular behavior.

### The SOX18^RaOp^ mutant protein interferes with the binding locations of wild-type SOX18 on a genome-wide scale

**Figure 3.**
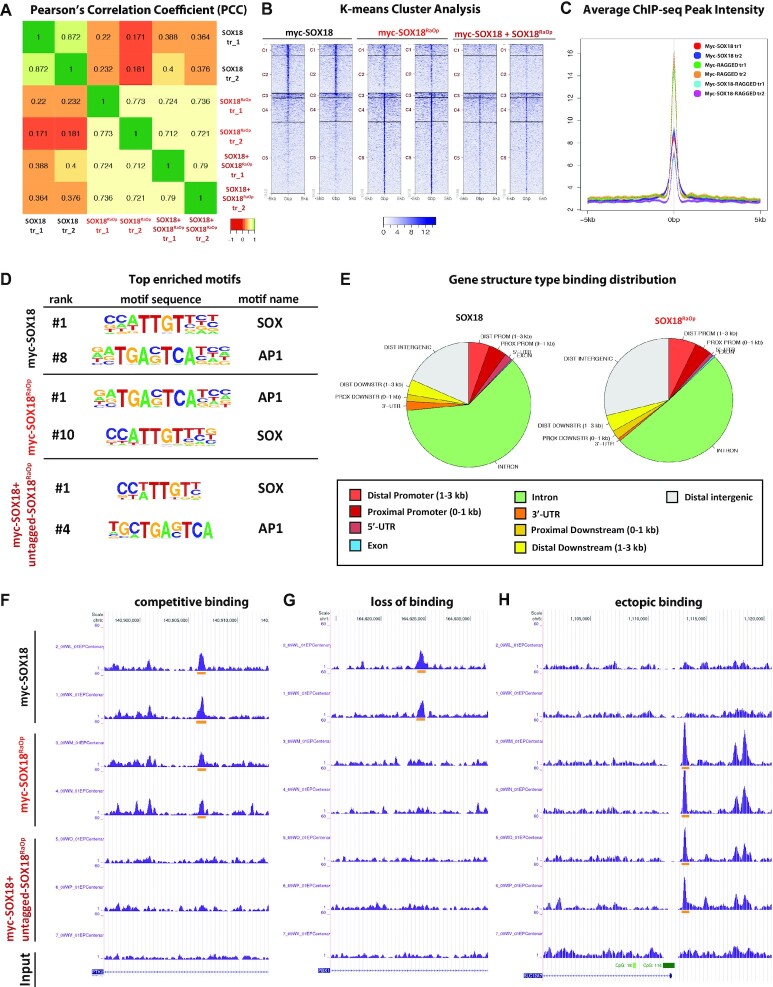
The SOX18^RaOp^ dominant-negative mutant protein competes with SOX18 for its genomic-binding sites and sequesters it at ectopic binding sites. (**A**) Pearson's Correlation Coefficient (PCC) analysis comparing the similarity in the genomic-binding profile of each sample in a pairwise manner. No correlation is shown in red (−1 PCC value) and a perfect correlation is shown in green (+1 PCC value). (**B**) *k*-means clustering of merged peaks into five different clusters (C1–C5) with the level of signal intensity shown in blue. (**C**) The average merged peak intensity for each condition, with myc-SOX18 replicate #1 shown in red, myc-SOX18 replicate #2 shown in dark blue, myc-SOX18^RaOp^ replicate #1 shown in green, myc-SOX18^RaOp^ replicate #2 shown in orange, myc-SOX18:untagged-SOX18^RaOp^ #1 shown in light blue and myc-SOX18:untagged-SOX18^RaOp^ #2 shown in purple. (**D**) Top enriched motifs for each condition. (**E**) Distribution of binding sites in relation to gene structure. (**F**) Representative ChIP-seq peaks demonstrating competition between SOX18 and SOX18^RaOp^ for the same binding site. (**G**) Representative ChIP-seq peaks demonstrating the loss of binding of SOX18 in the presence of SOX18^RaOp^. (**H**) Representative ChIP-seq peaks demonstrating ectopic binding of SOX18^RaOp^, and relocation of SOX18 to this site in the presence of SOX18^RaOp^.

To assess whether the observed perturbation in chromatin-binding dynamics translated to changes in genome-wide binding locations, we compared the binding profiles of SOX18 and SOX18^RaOp^, and assessed whether site recruitment changed for SOX18 in the presence of the dominant-negative mutant. In order to identify genomic-binding locations, we transfected HeLa cells in parallel with three different conditions: (i) myc-SOX18, (ii) myc-SOX18^RaOp^ and (iii) myc-SOX18 together with untagged-SOX18^RaOp^ in a 1:1 ratio, and performed ChIP-seq experiments using an anti-myc antibody (Figure 3). Each of these three conditions were performed in duplicate, giving a total of 6 samples.

First, to assess the reproducibility of the ChIP-seq dataset, and to compare the degree to which the binding locations of each of these three conditions differs, we performed a Pearson's correlation coefficient (PCC) test on each of the six samples, represented as a heatmap ranging from no correlation (−1, red) to perfect correlation (+1, green) (Figure 3A). Importantly, the PCC values for each of the duplicates is high (>0.7), indicating that the reproducibility of the ChIP-seq dataset is high. In contrast to this, the PCC values comparing myc-SOX18 to myc-SOX18^RaOp^ are very low (∼0.2), indicating that these two proteins have very different genomic-binding profiles. Further, the PCC values for myc-SOX18 compared to myc-SOX18:untagged-SOX18^RaOp^ is low (∼0.4), indicating that the presence of SOX18^RaOp^ significantly alters the genomic-binding locations of SOX18. This is supported by the high PCC value obtained for myc-SOX18^RaOp^ compared to myc-SOX18:untagged-SOX18^RaOp^ (∼0.7), indicating that SOX18^RaOp^ is strongly influencing this change in location of SOX18, giving it a more SOX18^RaOp^-like genomic binding pattern.

Second, as a further measure of data reproducibility, and to view these differences in genomic binding location on a global scale, we performed k-means clustering analysis which aligns the centre of the ChIP-seq peaks and sorts them into five clusters based on the closeness of the intensity of each ChIP-seq peak to the mean of each cluster (Figure 3B). Similar clustering profiles were generated for each of the duplicates, further validating the reproducibility of the ChIP-seq duplicates. In comparison to this, the clustering profiles of SOX18 and SOX18^RaOp^ were very different, indicating that these two proteins primarily bind different locations across the genome. Further, the presence of SOX18^RaOp^ appears to alter the genomic-binding locations of SOX18, by decreasing the binding of SOX18 to regions preferentially bound by SOX18 in the absence of SOX18^RaOp^ (Figure 3B, clusters C1 and C2), and increasing the binding of SOX18 to regions preferentially bound by SOX18^RaOp^ (Figure 3B, clusters C4 and C5).

Next, we quantified the average ChIP-seq peak intensity for each sample, which clustered across duplicates, and showed that SOX18^RaOp^ had the highest intensity (Figure 3C), in line with the hypothesis that SOX18^RaOp^ has higher affinity for chromatin.

Following on from the observation that SOX18 and SOX18^RaOp^ have significantly different genomic-binding locations and binding stabilities, we performed HOMER analysis to identify the most enriched binding motifs for each condition (Figure 3D). This analysis showed that the most enriched motif for SOX18 is a SOX site (motif rank #1 through to #7) followed by an AP-1 site at motif rank #8 (Figure 3D, Supplementary Table S3). These results show specificity of the ChIP-seq datasets and are in line with previously published ChIP-seq analysis performed in HUVECs for SOX18, whereby the top five motifs were SOX sites, followed by an enrichment for AP-1 motifs (ranks #6 through to #10) (34). Interestingly, this preference in motif binding was inverted for SOX18^RaOp^, with AP-1 instead being the most enriched motif (motif rank #1 through to #9), with a SOX site at motif rank #10. Further, the top 10 motifs bound by SOX18 in the presence of SOX18^RaOp^ were a mix of SOX and AP-1 sites, suggesting that SOX18^RaOp^ in part shifts the binding preference of SOX18 towards a SOX18^RaOp^-like binding profile.

Next, we assessed how this difference in motif binding preference of SOX18 and SOX18^RaOp^ altered the binding locations of SOX18 and SOX18^RaOp^ in relation to gene structure (Figure 3E). This showed that for wild-type SOX18, the vast majority of binding occurs within introns, followed by binding to distal intergenic regions. In comparison, SOX18^RaOp^ showed a significant shift away from intron binding towards more distal intergenic binding. To assess whether this differential binding involves regulatory elements of the genome, we compared the percentage of SOX18 and SOX18^RaOp^ binding sites that overlap with active and repressive histone marks (Supplementary Figure S6). By doing so we found that SOX18^RaOp^ had a higher percentage of binding to sites associated with active and repressive histone marks. This suggests that SOX18^RaOp^ may have the potential to repress genomic regions that would otherwise be active, as well as recruit SOX18 to transcriptionally silent regions of the genome.

After comparing differences in binding location of SOX18 and SOX18^RaOp^ at a genome-wide level, we focused our attention to different qualitative binding profiles occurring at individual genomic locations, (Figure 3F–H). Figure 3F shows an example of competitive binding whereby both SOX18 and SOX18^RaOp^ recognise the same binding location. As a consequence, this results in a decrease in the capability of SOX18 to bind this site in the presence of SOX18^RaOp^. Figure 3G shows an example of another type of interference mechanism, whereby there is a loss of SOX18 binding at a location that is not bound by SOX18^RaOp^. Potentially this loss in binding of SOX18 is due to sequestration by SOX18^RaOp^ either off the chromatin or to other locations. This is supported by Figure 3H, which shows ectopic binding of SOX18 only in the presence of SOX18^RaOp^, at a location recognised by SOX18^RaOp^ only. Overall, we found that that there is a dramatic reduction in the number of SOX18 binding events in presence of SOX18^RaOp^ (∼93% of SOX18 sites are lost) when compared to wild-type only conditions. This suggests that the cumulation of competition and sequestration events by SOX18^RaOp^ most likely represent the main form of interference impacting the spatial distribution of wild-type SOX18.

### The SOX18^RaOp^ mutant protein recruits SOXF factors to form non-functional complexes

**Figure 4.**
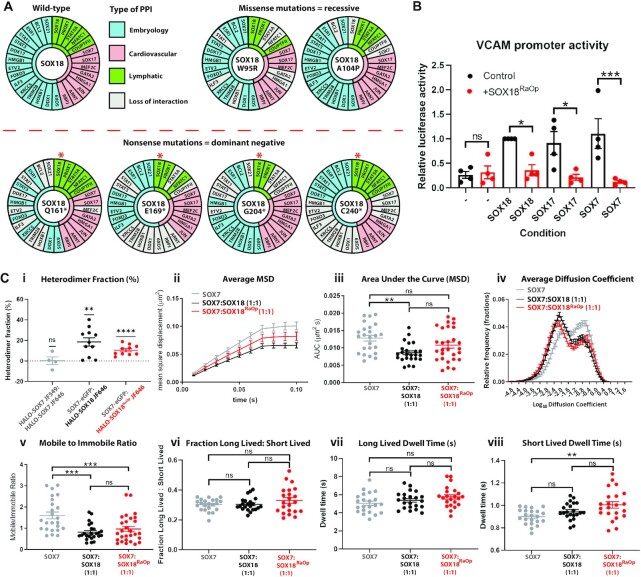
The SOX18^RaOp^ dominant-negative mutant protein recruits many SOX18 protein partners to form non-functional complexes. (**A**) AlphaScreen assay to assess pair-wise protein-protein interactions in presence of different SOX18 human mutations. Grey = loss of interaction. All dominant-negative mutants retain their ability to form a dimer with wild-type SOX18 (red asterisks). (**B**) Luciferase assay to measure VCAM1 promoter fragment transactivation (VCAM-Luc) in HeLa cells in the presence of SOX7, SOX17 and SOX18 and protein (black), and validation of the dominant-negative effect by adding SOX18^RaOp^ to SOX7, SOX17 and SOX18 (red). Values were made relative to the + VCAM1 + SOX18 condition which was set to 1. Values for the mean ± s.e.m. are shown. Statistical significance for pairwise comparisons was assessed using ANOVA, multiple comparisons were corrected using the Šidák correction. * *P* < 0.05, *** *P* < 0.001, ns = non-significant (*P* > 0.05). (**C**) (i) Quantification of the percentage of dimers formed for HALO-SOX7 using cRICS with two dyes (JF549 and JF646; grey), SOX7-eGFP:HALO-SOX18 JF646 (black) and SOX7-eGFP:HALO-SOX18^RaOp^ JF646 (red). Values for the mean ± s.e.m. are shown. *n* = 4, *n* = 11 and *n* = 10 (*N* = 3). Significance was assessed using a one-tailed *t*-test with a mu of 0. ** *P* < 0.01, **** *P* < 0.0001, ns = non-significant (*P* > 0.05). (ii–viii) Quantification of the dynamics of HALO-SOX7 (grey), HALO-SOX7 with untagged-SOX18 (black) in a 1:1 ratio and HALO-SOX7 with untagged-SOX18^RaOp^ in a 1:1 ratio (red). (ii) the average mean square displacement (MSD; μm^2^), (iii) the area under the curve (AUC) for the average mean square displacement (μm^2^s), (iv) the diffusion coefficient histogram for all cells (μm^2^/s) and (v) the mobile to immobile ratio. Threshold to classify mobile and immobile molecules is log_10_*D* = −1.5. Values for the mean ± s.e.m. are shown. Average number of trajectories: HALO-SOX7 = 2737, HALO-SOX7:SOX18 (1:1) = 2393, HALO-SOX7:SOX18^RaOp^ (1:1) = 2396. *n* = 24, *n* = 24 and *n* = 28 (*N* = 3). Data was log transformed for ANOVA analysis, raw is data displayed. Statistical significance was determined by a Tukey post-hoc test. ** *P* < 0.01 *** *P* < 0.001, ns = non-significant (*P* > 0.05). (vi) The fraction of long lived to short lived immobile events, duration of (vii) long-lived immobile and (viii) short-lived immobile events (s). Values for the mean ± s.e.m. are shown. *n* = 22, *n* = 22 and *n* = 22 (*N* = 3). Fraction long-lived:short-lived data was reciprocal (1/*Y*) transformed, and short-lived dwell time data was log transformed for ANOVA analysis, raw data is displayed. Statistical significance was determined by a Tukey post-hoc test. ** *P* < 0.01, ns = non-significant (*P* > 0.05).

The SOX18 TF has been reported to act via an array of multiple protein-protein interactions (PPIs) (34), thus as a broad spectrum mechanism for interference, we assessed whether SOX18^RaOp^ may have the potential to affect SOX18 PPIs. At first, we took advantage of the previous identification of the SOX18 interactome (34) (Figure 4A). We assessed whether different naturally occurring recessive and dominant-negative mutants mimicking those reported in Human (6,7,37–40) were able to retain their interaction with SOX18 protein partners by performing a protein–protein interaction assay using AlphaScreen technology. Here we show two main types of mutations – recessive mutations caused by the substitution of conserved residues within alpha helix 1 of the DNA-binding domain (W95R and A104P), and dominant-negative mutations caused by a premature truncation (Q161*, E169*, G204* and C240*). Even though some interactions are lost in some mutant conditions, a large number of protein partners are retained. Different subsets of protein partners are retained depending upon the extent of SOX18 protein truncation. Mutations G204* and C240* are the closest counterparts to SOX18^RaOp^ as the DNA-binding HMG domain and homodimerization DIM domain is left intact, and the C-terminal TAD domain is disrupted. This indicates that SOX18^RaOp^ has the potential to directly compete for SOX18 homodimer formation and protein partner recruitment to not only block SOX18 transcriptional activity but those of its interactors as well. Further, the difference in protein partner recruitment for different mutants would contribute to the variance in phenotype severity observed between mutants. Of note, AP-1 member JUN was recruited by wild-type SOX18 as well as every SOX18 mutant assessed here, which supports the ChIP-seq data showing an enrichment for AP-1 binding sites.

The current hypothesis on the dominant-negative mode of action of the SOX18^RaOp^ protein is its ability not only to disrupt SOX18 wild-type protein activity but more broadly to interfere with closely related SOXF family members (SOX7 and SOX17), which in turn inhibits any redundancy mechanism. As shown in Figure 4A, SOX7 and SOX17 are recruited by the majority of non-functional SOX18 mutants. This proposed molecular mechanism would explain why ragged mice exhibit severe vascular defects whereas the SOX18 knockout mice are devoid of cardiovascular defects (14,19,23) in certain genetic backgrounds. To validate this hypothesis, we performed a luciferase assay using the VCAM-1 promoter as a readout of the transcriptional capability of each SOXF member (Figure 4B). All SOXF members were able to activate the VCAM-1 promoter to varying degrees, however, were prevented from doing so by SOX18^RaOp^. The ability of SOX18 and SOX18^RaOp^ to recruit closely related SOX7 protein was further validated using cRICS, which showed SOX7/SOX18 (18.5%) and SOX7/SOX18^RaOp^ (10.5%) heterodimer formation (Figure 4Ci).

To analyze the level of interference of SOX18^RaOp^ on other SOXF members on a genome-wide scale, we performed SMT on HALO-SOX7 and HALO-SOX17 to quantify changes in their chromatin-binding dynamics in the presence of either untagged-SOX18 or untagged-SOX18^RaOp^ (Figure 4Cii-viii, Supplementary Figure S7 and Videos S5-8). We found that SOX18^RaOp^ significantly increased the chromatin-bound fraction and short-lived dwell time of SOX7 (Figure 4C). In parallel, SOX18^RaOp^ significantly increased the chromatin-bound fraction of SOX17 (Supplementary Figure S7). Therefore, this validates the formation of non-functional SOXF/SOX18^RaOp^ complexes on the chromatin, with an additional perturbation effect on the chromatin search pattern of SOX7.

### The SOX18^RaOp^ mutation perturbs the oligomeric state of SOX18

**Figure 5.**
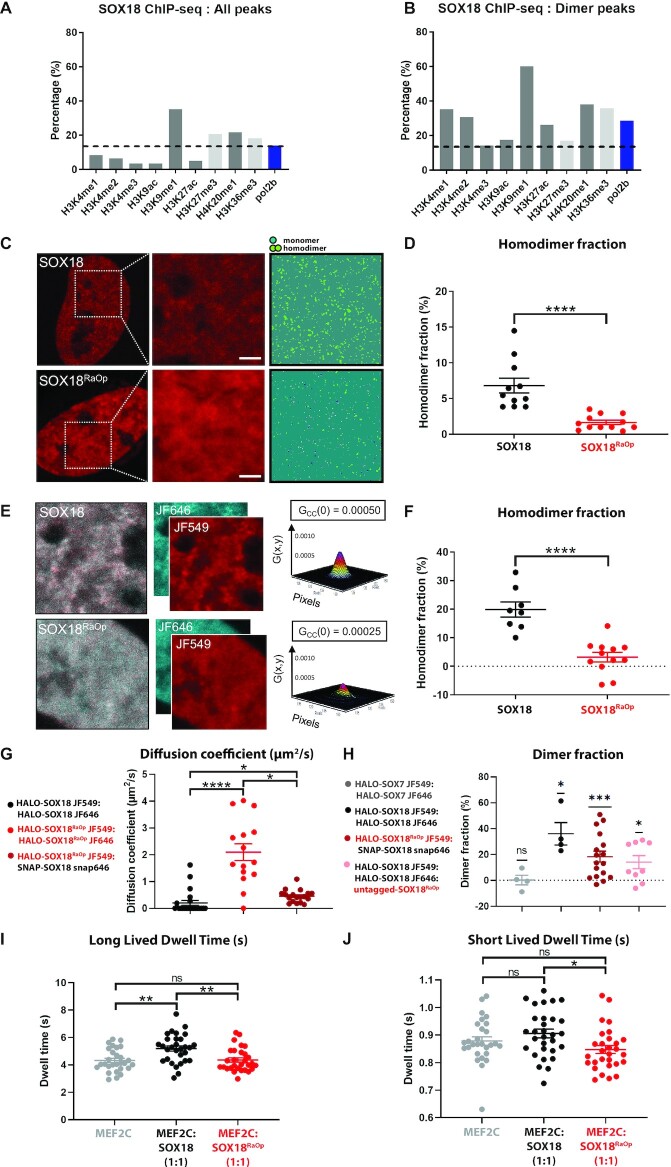
The SOX18^RaOp^ dominant-negative mutant protein has compromised homodimerization and impairs the scanning behavior of SOX18 dimer-specific protein partner MEF2C. (**A**) Overlay of SOX18 ChIP-seq peaks and (**B**) ChIP-seq peaks containing a dimer motif in HUVECs. Color code: activating histone marks (dark grey), repressive marks (light grey) and RNA polymerase II (blue). Percentage of ChIP-seq peaks that overlap with SOX18 ChIP-seq peaks by ≥ 50% (base-pairs). (**C**) N&B analysis of the oligomeric distribution of HALO-SOX18 and HALO-SOX18^RaOp^ (monomers/heterodimers = dark green and homodimers = light green). Scale bar = 2 μm. (**D**) Quantification of N&B analysis. HALO-SOX18 *n* = 11 and HALO-SOX18^RaOp^*n* = 12 (*N* = 2). Values for the mean ± s.e.m. are shown. Mann–Whitney *U*-test (two-tailed, unpaired). **** *P* < 0.0001. (**E**) cRICS analysis of (top row) two-color (JF549 and JF646 dyes) HALO-tagged SOX18 and (bottom row) HALO-tagged SOX18^RaOp^. The amplitude (*G*(0)) and decay (*D*) of the resulting 3D cRICS correlation profile was used to obtain the fraction and mobility of dimers, respectively. Scale bar = 2 μm. (**F**) cRICS quantification to obtain the fraction of homodimers for HALO-SOX18 (black) and HALO-SOX18^RaOp^ (red). *n* = 8 and *n* = 12 (*N* = 3). Values for the mean ± s.e.m. are shown. Mann–Whitney *U*-test (two-tailed, unpaired). **** *P* < 0.0001. (**G**) cRICS quantification to obtain the average diffusion coefficient of HALO-SOX18 homodimers (black), HALO-SOX18^RaOp^ homodimers (red) and SNAP-SOX18/HALO-SOX18^RaOp^ heterodimers (dark red). *n* = 22, *n* = 15 and *n* = 18. Values for the mean ± s.e.m. are shown. Statistical significance was determined by Kruskal-Wallis analysis. * *P* < 0.05, **** *P* < 0.0001. (**H**) cRICS quantification to obtain the fraction of HALO-SOX7 homodimers (grey), HALO-SOX18 homodimers (black), SNAP-SOX18/HALO-SOX18^RaOp^ heterodimers (dark red) and HALO-SOX18 homodimers in the presence of untagged-SOX18^RaOp^ (pink). *n* = 4, *n* = 4, *n* = 17 and *n* = 9. Values for the mean ± s.e.m. are shown. Significance was assessed using a one-tailed *t*-test with a mu of 0. * *P* < 0.05, *** *P* < 0.001, ns = non-significant (*P* > 0.05). (**I**) SMT to obtain the long-lived dwell times of HALO-MEF2C (grey), HALO-MEF2C:untagged-SOX18 (1:1) (black) and HALO-MEF2C:untagged-SOX18^RaOp^ (1:1) (red). Values for the mean ± s.e.m. are shown. *n* = 28, *n* = 30 and *n* = 29 (*N* = 3). Statistical significance was determined by a Tukey post-hoc test. ** *P* < 0.01, ns = non-significant (*P* > 0.05). (**J**) SMT to obtain the short-lived dwell times of HALO-MEF2C (grey), HALO-MEF2C:untagged-SOX18 (1:1) (black) and HALO-MEF2C:untagged-SOX18^RaOp^ (1:1) (red). *n* = 28, *n* = 30 and *n* = 29 (*N* = 3). Values for the mean ± s.e.m. are shown. Statistical significance was determined by a Tukey post-hoc test. * *P* < 0.05, ns = non-significant (*P* > 0.05).

One common PPI across all dominant-negative mutations is the wild-type SOX18 protein (Figure 4A, asterisks), which is consistently recruited by its mutant counterpart. This suggests that in presence of the SOX18^RaOp^ protein a mixture of different homo- and hetero-dimers are coexisting (SOX18/SOX18, SOX18/SOX18^RaOp^, SOX18^RaOp^/SOX18 and SOX18^RaOp^/SOX18^RaOp^), with a bias towards non-functional protein complexes. Previous work has reported that a functional feature of the SOX18 protein is its ability to form homodimers – a molecular state tightly associated with an endothelial-specific transcriptional signature (36). A key characteristic of TF activity is its ability to modulate mRNA transcription rate by communicating with basal transcriptional machinery. In order to further validate the functional relevance of the homodimer complex, we compared the overlap of total SOX18 and SOX18 homodimer only ChIP-seq peaks with active or repressive histone marks, and RNA polymerase II binding regions (Figure 5A and B) taking advantage of data sets from HUVECs generated by the ENCODE consortium (47,59–61). This showed an enrichment of the SOX18 ChIP-seq peaks which harbor a SOX dimer motif (Inverted repeat 5; IR 5 (36)) with a broad range of histone marks and RNA polymerase II. This observation indicates that the SOX18 homodimer localizes to transcriptionally active sites engaged in either activation or repression.

We next performed N&B and cross-RICS (cRICS) to quantify the spatial distribution and mobility of SOX18 homodimers. In order to label as many HALO-SOX18 and HALO-SOX18^RaOp^ molecules as possible whilst retaining fluctuations in fluorescence intensity as necessary for these techniques, we chose cells with low to medium expression levels, and used 1 μM of JF549 dye for N&B experiments, and 500 nM of JF549 and JF646 dyes for cRICS experiments as 1 μM total dye was shown to saturate HALO-SOX18 (Supplementary Figure S8). It is important to note that these dye concentrations are much higher than the one necessitated by SMT experiments (2 nM). To calibrate the brightness of the monomeric fraction we used HALO-SOX7 (Supplementary Figure S9) as no SOX7 homodimers were detected previously for this TF by AlphaScreen (36) or indirectly via ChIP-seq analysis since no IR5 dimer motif is enriched in SOX7 ChIP-seq peaks (34,36). This N&B approach revealed that even with low levels of homodimer detected at low HALO-SOX18 expression levels, SOX18 was found to form homodimer clusters (local enrichment of homodimers) throughout the nucleus (Supplementary Figure S9E). This clustering was found to increase further at higher levels of SOX18 expression, with the first evidence of a higher-order oligomeric form (more than three molecules in a complex) for SOX18 within homodimer clusters (Supplementary Figure S9E). By contrast, higher concentrations of SOX7 did not result in the formation of more homodimers as expected of a mono/heterodimeric protein (Supplementary Figure S9D). N&B analysis comparing the oligomeric profile of HALO-SOX18 and HALO-SOX18^RaOp^ showed that ∼7% of total SOX18 is SOX18 homodimers, whereas the homodimer population for SOX18^RaOp^ is significantly reduced at ∼2% of total SOX18^RaOp^ (Figure 5C and D).

To further validate the reduction of SOX18^RaOp^ homodimers observed in N&B, and investigate the mobility of this homodimeric population, we next performed cRICS analysis on HALO-SOX18 using two spectrally distinct Halo-tag fluorophores (JF549 and JF646) (Figure 5E–G). Co-movement of JF549 and JF646 tagged HALO-SOX18 dimers was observed by cRICS, thus validating the presence of the dimer in the N&B assay. Quantification of the two-color cRICS imaging experiment showed that ∼20% of SOX18 molecules are homodimers (Figure 5F), and this entire population exhibits a slow mobility (Figure 5G). As can be seen, cRICS was more sensitive toward SOX18 dimer detection than N&B, and this is likely due to the fact that the large population of monomers present (minimum 70%) reduces the apparent brightness recovered by N&B and the number of pixels classified as dimeric. N&B averages brightness being calculated in each pixel, therefore if there is a significant fraction of monomer present in a pixel the average brightness at that location will be pulled into the monomer cursor. Strikingly, the fraction of HALO-SOX18^RaOp^ homodimer was significantly depleted to ∼3%, confirming that SOX18^RaOp^ forms less homodimers than SOX18 (Figure 5F). Further, the mobility of SOX18^RaOp^ homodimers was significantly higher than the mobility of SOX18 homodimers (Figure 5G). This together with the observation that SOX18^RaOp^ has a higher chromatin-bound fraction and longer dwell time than SOX18 (Figure 1) indicates that the majority of SOX18^RaOp^ chromatin-binding occurs as a monomer, and that this interaction is more stable than that of the SOX18 wild-type.

The reduced HALO-SOX18^RaOp^ homodimer fraction observed using cRICS was further supported by a brightness aggregation assay performed using a temperature gradient. This approach showed that the human dominant-negative mutants akin to SOX18^RaOp^ due to truncations occurring within the transcriptional activation domain (G204* and C240*) required a higher temperature than SOX18 to form aggregates. This suggests a lesser potential for these mutants to homodimerize (Supplementary Figure S10). The opposite was shown for other mutant types (Q161* and E169*) which are shorter and have a higher propensity to form aggregates than the longer dominant-negative mutants and SOX18 wild-type. This observation indicates that protein length is likely a key contributing factor to the variation of the phenotypic outcome observed for different SOX18 mutants. As shown by AlphaScreen assay, wild-type SOX18 is recruited by all of its dominant-negative counterparts (Figure 4A). Therefore, the failure of SOX18^RaOp^ to homodimerize increases the availability of the mutant protein in the system, and likely skews protein assembly towards non-functional SOX18/SOX18^RaOp^ complexes. As a consequence, wild-type SOX18 function is further perturbed, leading to a more severe phenotype compared to SOX18^Ra^, SOX18^Ral^ and SOX18^RaJ^ which due to being shorter could have a higher propensity to self-associate rather than recruiting wild-type protein.

The validation of SOX18 dimer behavior was further controlled for by the use of a SOX18 mutant lacking the homodimerization domain (36) (HALO-SOX18^DIM^; Supplementary Figure S11, Videos S9 and S10). N&B confirmed that deletion of the DIM domain almost completely abolished SOX18 homodimer formation (Supplementary Figure S11A–D). Further, similar observations were made using the alpha helix 1 SOX18^AH1^ DNA-binding mutant (Supplementary Figure S11A–D, Videos S11 and S12). Collectively these results show that SOX18 dimers require DNA binding for their formation or maintenance, therefore suggesting that dimerization is primarily mediated via a cooperative mechanism. Analysis performed using the cRICS approach further validated these observations (Supplementary Figure S11E-I), where on average no homodimer was detected for the SOX18^DIM^ or SOX18^AH1^, indicating that the low level of residual homodimers observed by N&B were coincidental and likely to correspond to co-binding events whereby two molecules were juxtaposed on the DNA (Supplementary Figure S11I). SMT showed that there were less long-lived immobilization events than short-lived, and that both immobilization events occurred for a shorter total period of time (S11J). This observation suggests a change in genome scanning behavior and regulatory complex formation when the ability of SOX18 to form a dimer is compromised.

To validate the AlphaScreen data showing that SOX18 dominant-negative mutants recruit wild-type SOX18, we obtained a SNAP-SOX18 construct and performed cRICS on cells transfected with HALO-SOX18^RaOp^/JF549 and SNAP-SOX18/snap-646, using SOX7 as a monomeric control (Figure 5G and H). By doing so we detected a significant enrichment for SOX18/SOX18 homodimers and for SOX18/SOX18^RaOp^ heterodimers compared to a SOX7 monomeric control (Figure 5H). Further, the addition of untagged-SOX18^RaOp^ appears to reduce the percentage of SOX18 homodimers formed (14.1%) compared to the SOX18 only condition (36%), although this difference is non-significant suggesting that SOX18^RaOp^ may have a partial ability to compete with SOX18 for SOX18 dimer formation. We next set out to assess how the change in homodimerization behaviour of SOX18^RaOp^ would affect the recruitment of a SOX18 homodimer-specific protein partner.

### SOX18 homodimer-specific protein partner MEF2C is stabilised on the chromatin by SOX18, and destabilised by SOX18^RaOp^

Previously we have reported that MEF2C is preferentially recruited by a SOX18 homodimer (36). This SOX18 protein partner is essential for vascular development, with expression significantly enhancing the transcriptional capability of SOX18 (62). Further, previous SOX18 ChIP-seq analysis obtained in HUVECs identified that 5% of SOX18 ChIP-seq peaks also contain a MEF2C binding site (34). Due to the importance of MEF2C in modulating SOX18 activity during vascular development, and the observation that most SOX18 dominant-negative mutants retain binding to MEF2C (Figure 4A), we set out to assess the role of SOX18 in this complex, and whether SOX18^RaOp^ directly alters the molecular kinetics of MEF2C (Figure 5I and J, Supplementary Figure S12 and Videos S13 and S14) beyond the disruption of the chromatin-binding dynamics of the SOXF group. Measuring MEF2C behavior by SMT in the presence of SOX18 revealed an increase in the long-lived dwell times (Figure 5I) but did not change the chromatin-bound fraction (Supplementary Figure S12), validating the recruitment of MEF2C and indicating that the SOX18 homodimer plays a role in stabilizing MEF2C on target sites. In comparison to this, SOX18^RaOp^ did not increase the long-lived dwell time of MEF2C, and rather decreased its short-lived dwell time, indicating that SOX18^RaOp^ fails to stabilize MEF2C on target sites, and disrupts its search for target sites along non-specific chromatin. This observation aligns with the finding that SOX18^RaOp^ homodimers are not stably bound to the chromatin like SOX18 homodimers (Figure 5G).

This study establishes a set of molecular rules which are necessary to drive genome-wide perturbation in the context of transcription factor dominant-negative mutation and therefore instruct the phenotypic outcome of a genetic disease. Hallmarks of the dominant-negative mechanism are characterized by the capacity to disable key features of SOX18 activity: (i) via interference with chromatin sampling behavior through increased dwell times of non-functional complexes at target sites, (ii) compromised oligomerization, (iii) broadly poisoning the SOX18 interactome which in turn impacts other TF networks and (iv) by disrupting the genome-wide binding profile and causing ectopic or lack of specific site recruitment.

## DISCUSSION

**Figure 6.**
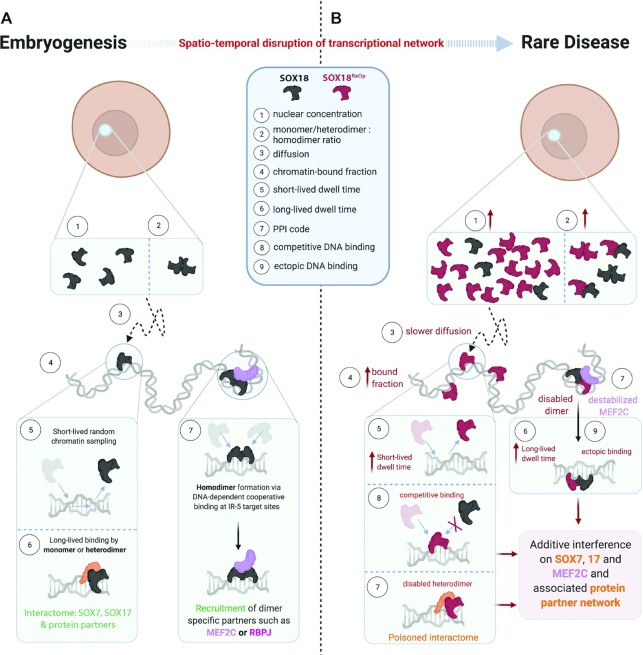
Components that make up the behavior of SOX18 in a physiological state, and how these regulatory layers are impacted by a dominant-negative SOX18 mutant to cause potent transcriptional repression. Created with BioRender.com (**A**) SOX18 transcription factor (black) behavior in a normal physiological state. (1) SOX18 is present at physiological levels within the nucleus (2) and exists in different oligomeric states. (3) SOX18 exhibits diffusion throughout the nucleus in between immobile events on the chromatin. (4) The SOX18 chromatin-bound fraction represents around 20% of its population. (5) SOX18 dwells for a short period of time (less than 1 s) on non-specific sites as it samples the chromatin to identify target sites. (6) SOX18 binds to target sites where it remains for longer (a few seconds). (7) SOX18 homodimer formation and maintenance are DNA-dependent via cooperative binding to recruit homodimer-specific partners such as MEF2C or RBPJ. (**B**) SOX18^RaOp^ (red) is a dominant-negative mutant of SOX18 and exhibits aberrant behaviors of steps 1–7. Further, SOX18^RaOp^ (8) competes with SOX18 for genomic-binding sites and (9) sequesters SOX18 at ectopic binding sites. Collectively, these behavioral changes lead to large scale transcriptional dysregulation. The interference of SOX protein partners by SOX18^RaOp^ is additive, hence disrupting multiple regulatory networks.

In this study, we uncovered several core biophysical properties that define how the key vascular and hair follicle regulator SOX18 navigates the genome. Further, we describe the multifaceted way by which a dominant-negative SOX18 mutant interferes with its wild-type counterpart to perturb this search pattern on the chromatin while poisoning other TF networks via protein–protein interactions (Figure 6). Our findings explain at the molecular level on a genome-wide scale the etiology of a rare disease which is underpinned by a non-functional TF.

The chromatin-binding stability of a TF is dictated by the protein-protein, protein-DNA and protein-RNA interactions that it forms (1,2). In support of this DNA-binding and homodimerization SOX18 mutants (SOX18^AH1^ and SOX18^DIM^) showed a significant reduction in their chromatin-binding capabilities. Surprisingly, the opposite effect was observed for SOX18^RaOp^, indicating that the chromatin-binding behavior of this mutant is likely due to an increase in chromatin-binding stability. In further support of this, despite being expressed at comparable levels with SOX18 throughout the entire cell, more SOX18^RaOp^ was retained within the nucleus. This increased stability may be explained by the change in the iso-electric point of SOX18^RaOp^ (i.e. 10.4) which is much higher than its wild-type counterpart (i.e. 7.3) (63). The DNA-binding HMG domain is already extremely positively-charged in order to bind negatively-charged DNA efficiently, so here we proposed that overall SOX18^RaOp^ acts as a chromatin ‘magnet’, which in turn causes an increase in the number of sites recognized as ‘specific’ and its dwell times. The scrambling and premature truncation of the transactivation domain of SOX18^RaOp^ causes the overall charge to become skewed in favour of the positively charged DNA-binding HMG domain rich in high pKA amino acids, therefore likely increasing its affinity for negatively-charged DNA.

Altering the DNA-binding stability of a TF has been shown to modulate the type of DNA motif that it recognises (64). This has been observed for MITF, where a mutation mimicking acetylation in the DNA-binding domain that reduces its overall binding affinity caused a change in binding behaviour, shifting preference from low affinity degenerated sites to high affinity consensus sites. Motif enrichment analysis reveals that SOX18 preferentially binds to SOX sites, and to a lesser extent to AP-1 sites. By contrast, SOX18^RaOp^ displays an inverted binding profile enriched in AP-1 sites compared to SOX motifs. This shift in binding site preference likely causes SOX18^RaOp^ to disable SOX18 through a number of mechanisms, including sequestration of SOX18 away from its usual binding sites, recruitment of SOX18 to ectopic sites, in addition to competitive binding for a subset of sites that are recognised by both wild-type and mutant proteins. Further, the increased affinity of SOX18^RaOp^ for the AP-1 binding motif is likely to amplify the dominant-negative effect and is further supported by the fact that all Ragged protein mutants are able to recruit c-JUN TF, a member of the AP-1 family. Previously, AP-1 proteins have been shown to maintain chromatin accessibility, with the majority of c-JUN binding sites (∼90%) found in open chromatin regions (65). This positioning of AP-1 sites in open chromatin regions may explain the shift in SOX18^RaOp^ towards binding such regions. As a result, the redistribution of SOX18^RaOp^ may have implications on genome-wide chromatin accessibility.

Through some unknown mechanism, SOX18^RaOp^ interferes with the ability of other SOXF members (SOX7 and SOX17) to rescue the phenotype. We hypothesized that this interference may be reflected by changes in their chromatin-binding dynamics in the presence of SOX18^RaOp^ as compared to SOX18. We observed that SOX18^RaOp^ altered the chromatin-binding behavior for both SOXF factors, by significantly increasing the percentage of non-functional complexes on the chromatin, suggesting that SOX18^RaOp^ has the ability to compete with SOX18 for SOXF factor recruitment. This supports the PPI data showing that SOXF factors are recruited by SOX18 and the majority of SOX18 mutants assessed here.

To investigate a potential interference with a shared protein partner, we assessed the ability of SOX18 and SOX18^RaOp^ to alter the chromatin-binding dynamics of MEF2C. Rather unexpectedly, SOX18 increased MEF2C stability on target sites, whereas SOX18^RaOp^ destabilized MEF2C on both specific target sites, and during its search along non-specific chromatin sites. MEF2C has previously been reported to be preferentially recruited by a SOX18 homodimer (36), and, since SOX18^RaOp^ has a significant reduction in homodimers, and these homodimers have higher mobilities, this suggests that it would be unable to efficiently stabilize MEF2C on active chromatin sites. Importantly, this molecular scenario is not at play in the context of SOX18 targeted gene disruption whereby a lack of SOX18 does not interfere directly with the search pattern mechanism of MEF2C, hence limiting its range of interference.

It remains unclear as to why some interactions are strengthened and others weakened, although given that different PPIs involve different protein subdomains this is not entirely unexpected. The mutant protein retains multiple protein partners, suggesting that the observed negative effect on transcription could potentially be amplified via interference with multiple regulatory hubs. This hypothesis is supported by the observation that the aggregation profile and subsequent interactome of the different dominant-negative human mutations correlates with disease phenotype severity. The longer the mutant protein, the more severe the phenotype, in other words as the mutant protein gets shorter it loses the ability to poison other protein complexes.

Although we have made considerable progress towards uncovering the molecular mechanisms that underpin a genetic disorder, it remains unknown as to how these changes directly relate to the mutant phenotypes observed in humans and mice. Genetic studies have shown that SOX18 directly controls the transcription of other key genetic pathways, such as Notch1 for arterial specification (25,26), or Prox1 for lymphangiogenesis (15). Based on this, the question posed could be answered by directly measuring at a single locus (at a particular enhancer or promoter of Notch1 or Prox1 for instance) the chromatin-binding dynamics of SOX18 verses SOX18^RaOp^. At present, technological limitations relating to the sparse labelling approach required to perform SMT do not allow for this type of resolution. The number of events at a single locus is simply too low and not compatible with a fast acquisition rate (10 ms).

Prior to this study, only mathematical models to describe the broad spectrum interference of a dominant-negative TF have been developed (5). These models used two main variables: the level of allelic expression combined with the genetic configuration (homozygous or heterozygous). Here we reveal eight quantifiable components: nuclear concentration, the ratio of monomers/heterodimers to homodimers, the rate of protein diffusion, the chromatin-bound fraction, dwell times at specific target sites, dwell times at non-specific sites, protein partner recruitment and TF binding motif selection. Further, we show that not only does the dominant-negative protein interfere with its wild-type counterpart, but it also interferes with the behavior of other classes of TF such as MEF protein. This extends the interference mechanism beyond just perturbing its own regulatory network, since the mutant affects the regulatory hubs belonging to its protein partners. This level of interference was not appreciated before and even less so demonstrated at the experimental level.

The question arises as to how applicable our model is to other TF families. Here we propose two criteria to predict whether mutations would cause any TF to broadly interfere with transcription in a SOX18^RaOp^-like fashion. Firstly, the dominant-negative mutation should leave the DNA-binding domain intact and disrupt the transactivation domain to remove its transcriptional capability whilst still enabling key protein partner recruitment. Secondly, the wild-type form should form DNA-dependent dimers. Based on these assumptions, we believe that this model will not only apply to other SOX members with dominant-negative TF counterparts, but also to TFs of other families that satisfy these criteria.

In conclusion, by combining imaging techniques with genomics and proteomics assays, we were able to quantitate the effects of a dominant-negative TF on a genome-wide scale. We demonstrate that this broad interference is likely mediated by multiple biophysical parameters that directly relate to wild-type protein activity but also expand to other regulatory hubs engaged via PPIs. Looking ahead with the advent of new ways to visualize specific genomic locations or measure transcription rate in real time, the next step will be to correlate changes in TF activity measured in real time with their corresponding transcriptional output. This type of approach combined with multi-omics analysis will better our understanding of the molecular basis of gene regulation.

## DATA AVAILABILITY

All single molecule tracking (SMT), number and brightness (N&B) and cross-raster image correlation spectroscopy (cRICS) raw data has been deposited at DataDryad and made publicly available. Further, ChIP-seq raw data has been deposited at ArrayExpress and made publicly available.

Fast (20 ms for 6000 frames) and slow (500 ms for 500 frames) raw data used for SMT analysis can be accessed using the DataDryad link: https://doi.org/10.5061/dryad.xsj3tx9fp

Examples of SMT movies for each condition acquired using fast (20 ms for 6000 frames) and slow (500 ms for 500 frames) tracking methods can be accessed using the DataDryad link: https://doi.org/10.5061/dryad.8w9ghx3n7

Raw data used for N&B and cRICS analysis can be accessed using the DataDryad link: https://doi.org/10.5061/dryad.h9w0vt4gn.

ChIP-seq raw data have been deposited in the ArrayExpress database at EMBL-EBI (www.ebi.ac.uk/arrayexpress) under accession number E-MATB-10609, and can be accessed using the link: https://www.ebi.ac.uk/arrayexpress/experiments/E-MTAB-10609.

ChIP-seq tracks can be viewed in UCSC Genome Browser by entering the following URLs into the load custom tracks option of the browser:

track type=bigWig name=1_09WK_01EPCentenary_Myc-SOX18_tr1_Myc-tag_hg38_i67_uniqnorm description=1_09WK_01EPCentenary_Myc-SOX18_tr1_Myc-tag_hg38_i67_uniqnorm graphType=bar bigDataUrl=ftp://gp_mfrancois:%26%6d%5f%4a%32%2d%52%4b@ftp.activemotif.com/BW/1_09WK_01EPCentenary_Myc-SOX18_tr1_Myc-tag_hg38_i67_uniqnorm_signal.bw db=hg38 visibility=2 color=102,24,202
track type=bigWig name=2_09WL_01EPCentenary_Myc-SOX18_tr2_Myc-tag_hg38_i68_uniqnorm description=2_09WL_01EPCentenary_Myc-SOX18_tr2_Myc-tag_hg38_i68_uniqnorm graphType=bar bigDataUrl=ftp://gp_mfrancois:%26%6d%5f%4a%32%2d%52%4b@ftp.activemotif.com/BW/2_09WL_01EPCentenary_Myc-SOX18_tr2_Myc-tag_hg38_i68_uniqnorm_signal.bw db=hg38 visibility=2 color=102,24,202
track type=bigWig name=3_09WM_01EPCentenary_Myc-RAGGED_tr1_Myc-tag_hg38_i69_uniqnorm description=3_09WM_01EPCentenary_Myc-RAGGED_tr1_Myc-tag_hg38_i69_uniqnorm graphType=bar bigDataUrl=ftp://gp_mfrancois:%26%6d%5f%4a%32%2d%52%4b@ftp.activemotif.com/BW/3_09WM_01EPCentenary_Myc-RAGGED_tr1_Myc-tag_hg38_i69_uniqnorm_signal.bw db=hg38 visibility=2 color=102,24,202
track type=bigWig name=4_09WN_01EPCentenary_Myc-RAGGED_tr2_Myc-tag_hg38_i70_uniqnorm description=4_09WN_01EPCentenary_Myc-RAGGED_tr2_Myc-tag_hg38_i70_uniqnorm graphType=bar bigDataUrl=ftp://gp_mfrancois:%26%6d%5f%4a%32%2d%52%4b@ftp.activemotif.com/BW/4_09WN_01EPCentenary_Myc-RAGGED_tr2_Myc-tag_hg38_i70_uniqnorm_signal.bw db=hg38 visibility=2 color=102,24,202
track type=bigWig name=5_09WO_01EPCentenary_Myc-SOX18-RAGGED_tr1_Myc-tag_hg38_i71_uniqnorm description=5_09WO_01EPCentenary_Myc-SOX18-RAGGED_tr1_Myc-tag_hg38_i71_uniqnorm graphType=bar bigDataUrl=ftp://gp_mfrancois:%26%6d%5f%4a%32%2d%52%4b@ftp.activemotif.com/BW/5_09WO_01EPCentenary_Myc-SOX18-RAGGED_tr1_Myc-tag_hg38_i71_uniqnorm_signal.bw db=hg38 visibility=2 color=102,24,202
track type=bigWig name=6_09WP_01EPCentenary_Myc-SOX18-RAGGED_tr2_Myc-tag_hg38_i74_uniqnorm description=6_09WP_01EPCentenary_Myc-SOX18-RAGGED_tr2_Myc-tag_hg38_i74_uniqnorm graphType=bar bigDataUrl=ftp://gp_mfrancois:%26%6d%5f%4a%32%2d%52%4b@ftp.activemotif.com/BW/6_09WP_01EPCentenary_Myc-SOX18-RAGGED_tr2_Myc-tag_hg38_i74_uniqnorm_signal.bw db=hg38 visibility=2 color=102,24,202
track type=bigWig name=7_09WV_01EPCentenary_HeLa_Pooled_Input_hg38_i75_uniqnorm description=7_09WV_01EPCentenary_HeLa_Pooled_Input_hg38_i75_uniqnorm graphType=bar bigDataUrl=ftp://gp_mfrancois:%26%6d%5f%4a%32%2d%52%4b@ftp.activemotif.com/BW/7_09WV_01EPCentenary_HeLa_Pooled_Input_hg38_i75_uniqnorm_signal.bw db=hg38 visibility=2 color=102,24,202

## Supplementary Material

gkab820_Supplemental_Files
