# Supplementary material for: A dominant-negative SOX18 mutant disrupts multiple regulatory layers essential to transcription factor activity
Source: Nucleic Acids Res. 2021 Sep 27;49(19):10931–55. doi: 10.1093/nar/gkab820 (PMC8565327; doi:10.1093/nar/gkab820)
Supplement: gkab820_Supplemental_Files [file gkab820_supplemental_files.zip › NAR Supplementary Data PDF.pdf]

## SUPPLEMENTARY TEXT

### Further information on differences between SMT algorithms

To assess whether the discrepancy in the estimated diffusion coefficient for SOX18 obtained in this manuscript, and SOX2 in the Chen *et al.* manuscript (2) was due to biological differences, we performed SMT on HALO-SOX2, analysed the data using PalmTracer and an alternative track-based method: the SLIMfast analysis pipeline developed in MATLAB for the aforementioned SOX2 paper, and compared it to HALO-SOX18 (Figure S5A). By doing so we found that SOX2 and SOX18 exhibited similar chromatin-binding behaviours, therefore confirming that the discrepancy observed was not due to a biological difference. This was somewhat expected as SOX2 and SOX18 are members of the same TF family and share a highly conserved DNA-binding domain. Rather, we found that there was a significant difference between the estimates produced by PalmTracer and SLIMfast using the same datasets. Although the diffusion coefficient frequency peak for the bound population remained at a similar $\log_{10}$ diffusion coefficient value for SOX2 and SOX18 across both analyses, the diffusion coefficient peak for the unbound population was significantly shifted to the right for the SLIMfast analysis, indicating that SLIMfast estimates higher diffusion coefficients for unbound molecules. This shift in the unbound fraction towards the right has a knock-on effect on the bound fraction leading to a downwards shift in the SLIMfast data.

To ensure that the significant difference we observed in the chromatin-binding behaviours of SOX18 and $SOX18^{RaOp}$ (Fig. 1) was genuine and could be replicated across different SMT analysis software, we also assessed the diffusion coefficients and bound fractions of SOX18 and $SOX18^{RaOp}$ using SLIMfast (Figure S5B, Table S2). This method validated the significant difference in the chromatin-binding behaviours of SOX18 and $SOX18^{RaOp}$, as evident by a higher chromatin-bound peak in the diffusion coefficient histogram for $SOX18^{RaOp}$ as compared to SOX18, and the peak for the unbound fraction for $SOX18^{RaOp}$ being further to the left than SOX18. These differences lead to a significantly higher bound fraction, and a lower unbound diffusion coefficient for $SOX18^{RaOp}$ (Table S2), which correlates with the results obtained by PalmTracer.

Further, to account for differences in analysis that may be due to using track-based methods, we also tested a jump distance-based analysis software written in MATLAB called Spot-On (34) (Fig. S5C and D, and Table S2). We assessed Spot-On using both PalmTracer and SLIMfast analysis files as inputs, and fit cells to both 2-component (1 bound and 1 unbound fraction) and 3-component models (1 bound and 2 unbound fractions). First, we used the PalmTracer analysis files as input. By doing so we found that 64 % of SOX18 cells and 47 % of $SOX18^{RaOp}$ cells were best fit to a 3-component model. Both 2- and 3-component models gave a significantly higher bound fraction for $SOX18^{RaOp}$ as compared to SOX18, and a significantly lower diffusion coefficient for the unbound fraction(s) of $SOX18^{RaOp}$ compared to SOX18, therefore supporting the results obtained by PalmTracer and SLIMfast. Next, we compared this to Spot-On analysis using SLIMfast analysis files as input. This showed that 55 % of SOX18 cells and 68 % $SOX18^{RaOp}$ cells were best fit to a 3-component model. Although the difference

in the bound fraction of SOX18 and SOX18$^{RaOp}$ was non-significant using the 2- and 3-component models, the trend was the same as the other analysis methods, with SOX18$^{RaOp}$ having a higher average bound fraction compared to SOX18. Further, the diffusion coefficient for the unbound fraction of SOX18$^{RaOp}$ using 2-component analysis, and the diffusion coefficient for one of the unbound fractions for SOX18$^{RaOp}$ using 3-component analysis, was significantly lower than that of SOX18. Of note, the diffusion coefficients produced by Spot-On using SLIMfast analysis files as input were closest to those estimated by PalmTracer.

In conclusion, we found that the algorithms used are mainly responsible for giving different results with a major difference in the unbound fraction which explains discrepancy with previous publications regarding the diffusion coefficients. This is likely due to these SMT algorithms taking different approaches towards avoiding mistracking, with some being more conservative than others leading to lower estimated diffusion coefficients, and others prioritizing absolute values leading to higher estimated diffusion coefficients. Here, our main focus is whether the difference observed between wild-type SOX18 and its mutant counterpart SOX18$^{RaOp}$ is genuine. Regardless of the algorithm used, SOX18$^{RaOp}$ had a higher chromatin-bound fraction than SOX18, and SOX18 had a higher diffusion coefficient for the unbound fraction compared to SOX18$^{RaOp}$, therefore indicating that this difference is indeed genuine.

## SUPPLEMENTARY FIGURES

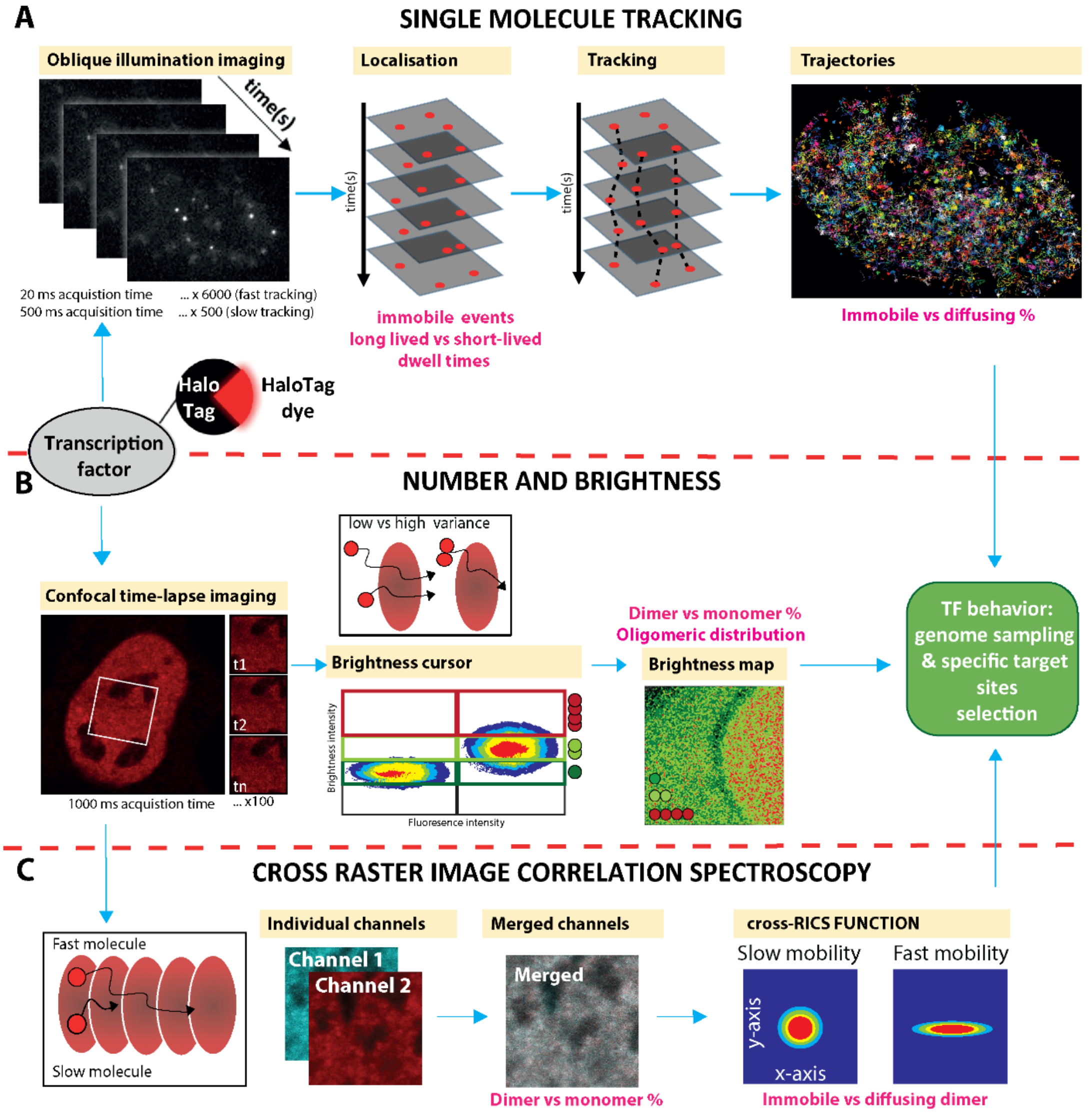


**Figure S1. Combination of single molecule-based assays in live cells to obtain the chromatin-binding dynamics, oligomeric distribution and immobile homodimer fraction of a transcription factor in order to uncover core components of its behavior.**

Halo-tag labelling technology was used for all experiments.

**(A) Single molecule tracking (SMT).** A series of images is taken using oblique illumination in order to penetrate the nucleus with a thin beam, greatly reducing background fluorescence and obtaining single molecule resolution. **Fast tracking SMT (20 ms acquisition times, 6000 frames):** in order to obtain the trajectories of each molecule, an acquisition time of 20 ms is used to capture diffusing molecules with faster mobilities in addition to immobile molecules. The location of each of the molecules in each of the frames is located (localization) and connected between frames (tracking) to obtain the trajectories of each molecule. Quantification of the trajectories gives immobile and mobile diffusing fractions. **Slow tracking SMT (500 ms acquisition times, 500 frames):** within the immobile fraction of molecules,

subpopulations of molecules with different dwell times exist. An acquisition time of 500 ms is used to decrease the signal of mobile diffusing molecules and enhance visualization of immobile molecules to enable quantification of their dwell times and percentages.

**(B) Number and brightness (N&B):** A time series of 100 frames (256 x 256 pixel frame size) is acquired in a zoomed in region of interest (white box outline) that is raster scanned with a 12.5 μs pixel dwell time. This scan rate (1 frame per second) focuses on molecules with moderate mobility. For each pixel in this region of interest, the change in fluorescence intensity over time is measured and converted to into a molecular brightness value, which is indicative of the average oligomeric state of the molecules present in that pixel. A homodimer is twice as bright as a monomer or heterodimer (which cannot be distinguished here due to a single tag being used) and a higher-order oligomer is brighter than the dimeric state. Monomeric brightness is calibrated by N&B analysis of a transcription factor that does not form detectable homodimers and is used to extrapolate the brightness of dimers and higher order oligomers. This calibration thus enables definition of cursors that colour code brightness maps and show the spatial distribution of the different oligomeric states of an oligomeric TF throughout the cell. From the brightness maps it is possible to infer the ability for a TF to form homodimers and higher-order oligomers, and the ability of these species to generate clusters (higher local concentration of homodimers and higher-order oligomers). Shown in this example are monomers in dark green, homodimers in light green and higher order oligomers in red.

**(C) Cross-raster image correlation spectroscopy (cRICS).** Like N&B, for cRICS, a time series of 100 intensity frames (256 x 256 pixel frame size) in a selected in region of interest (white box outline) is acquired using a 12.5 μs pixel dwell time. However, unlike N&B, for cRICS the acquisition is recorded in two channels and it must be recorded at a zoom that ensures the laser point spread function is oversampled (10.6 μm frame gives rise to a 41 nm pixel size). The fluorescence intensity in each frame in the acquisition is then spatially correlated within each channel via application of the RICS function, as well as spatially cross-correlated between channels via application of the cRICS function. In each instance, the average spatial correlation function is calculated across the 100 frames and fit to a one or two component diffusion model. Comparison of the amplitude recovered from fitting the average cRICS versus RICS functions enables the fraction of TF heterodimer present to be quantified (or TF homodimer in the instance two spectrally distinct Halo-tag dyes are employed to label a single TF). Comparison of the diffusion coefficient recovered from fitting the average cRICS versus RICS functions enables the mobility of the TF monomer versus heterodimer (or homodimer) to be recovered.

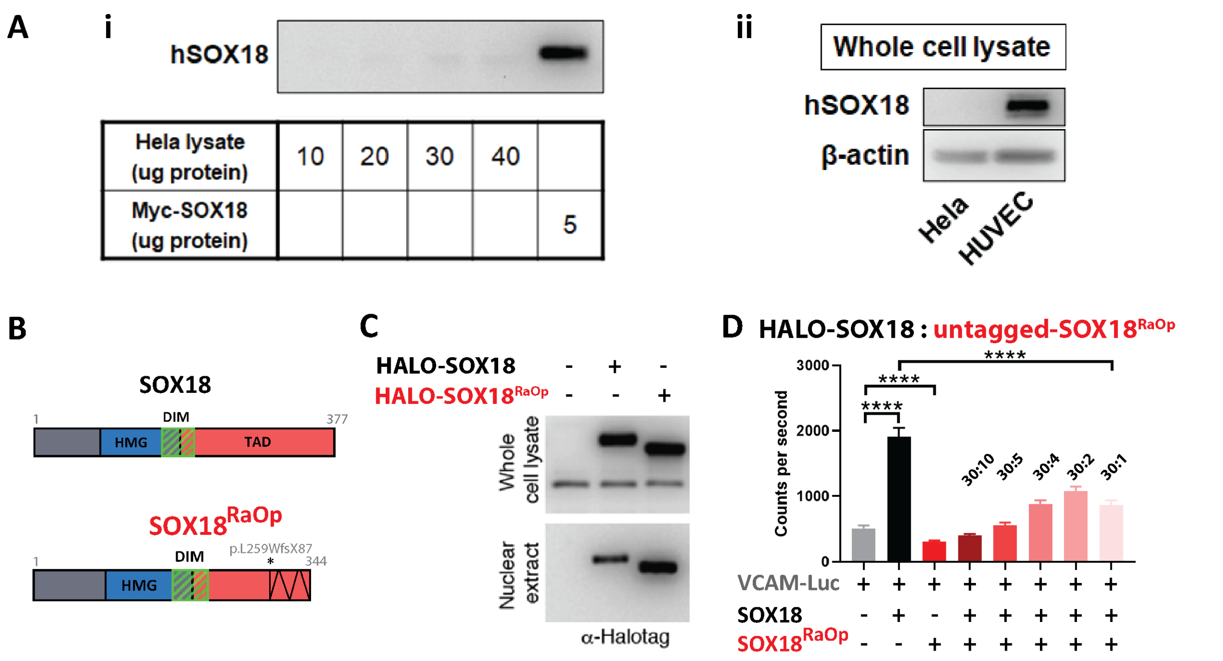


**Figure S2. HeLa cells show no detectable endogenous levels of SOX18 protein, the Ragged Opossum (SOX18$^{RaOp}$) mutation does not interfere with SOX18 protein expression, and the HaloTag does not interfere with the ability of SOX18 to activate gene transcription, or the ability of SOX18$^{RaOp}$ to repress SOX18.**

**(A)** Western blot analysis using an anti-human SOX18 antibody for human SOX18 (hSOX18) detection. **(i)** Lanes 1-4: 10, 20, 30 and 40 µg total protein from HeLa cell lysate. Lane 5: 5 µg of transfected human Myc-SOX18 protein. **(ii)** Western blot analysis on whole cell lysate from Hela cells (lane 1) and human umbilical venous endothelial cells (HUVECs; lane 2), with beta-actin used as a control.

**(B)** Representation of SOX18 (top) and SOX18$^{RaOp}$ (bottom) protein domains. SOX18 and SOX18$^{RaOp}$ both share the same DNA-binding and bending HMG domain (blue) and homodimerization domain (DIM; green diagonal stripes), however SOX18$^{RaOp}$ has a point mutation (c.775delC; asterisk) in its C-terminal transactivation domain (red) which scrambles the rest of the domain (black zigzag) before resulting in a premature stop codon.

**(C)** Western blot analysis on whole cell extracts from control conditions (empty vector) or transfected with HALO-SOX18 or HALO-SOX18$^{RaOp}$ show a similar expression level of protein. Western-blot analysis from nuclear extracts (ctrl, HALO-SOX18 or HALO-SOX18$^{RaOp}$) show an increase in the mutant protein. A halo-tag antibody was used for HALO-SOX18 or HALO-SOX18$^{RaOp}$ detection.

**(D)** Luciferase assay to measure VCAM1 promoter fragment transactivation (VCAM-Luc) in the presence of HALO-SOX18 (black) and SOX18$^{RaOp}$ (red) protein. Validation of the dominant-negative effect using different ratios of HALO-SOX18:SOX18$^{RaOp}$ (30:10, 30:5, 30:4, 30:2 and 30:1; dark-red to light-red gradient). VCAM-Luc transactivation by SOX18 and SOX18$^{RaOp}$ was performed in COS-7 cells, and is measured in counts per second, luciferase, arbitrary unit. A robust regression and outlier removal

(ROUT) outlier test using default settings (Q = 1 %) in GraphPad Prism was performed to identify and remove outliers. Values for the mean ± s.e.m. are shown. Data was log transformed for ANOVA analysis, raw data is displayed. Statistical significance was determined by a Tukey post-hoc test. **** $P<0.0001$.

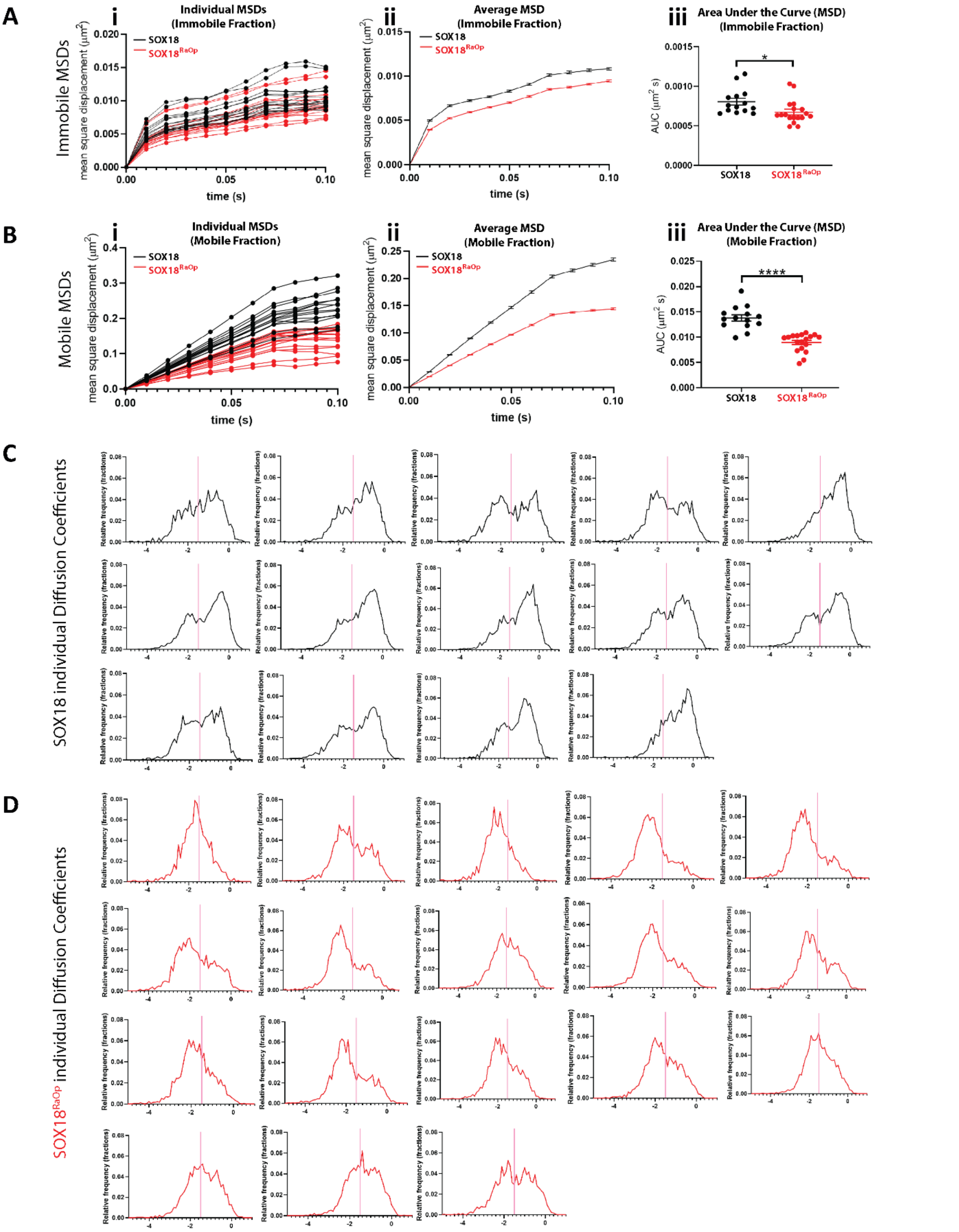


**Figure S3. Average MSDs with separated mobile and immobile fractions and diffusion coefficient histograms for each cell for HALO-SOX18 and HALO-SOX18$^{RaOp}$.**

**(A)** The average mean square displacement (MSD) for HALO-SOX18 (black) and HALO-SOX18$^{RaOp}$ (red) separated into immobile fractions for each cell **(i)** and for each sample **(ii)**. **(iii)** Quantification of

the average MSD for immobile fractions for each cell is shown as area under the curve of the MSD. Values for the mean ± s.e.m. are shown. HALO-SOX18 n = 14 and HALO-SOX18$^{RaOp}$ n = 18 (N = 3). t-test (two-tailed, unpaired). * P <0.05.

**(B)** The average mean square displacement (MSD) for HALO-SOX18 (black) and HALO-SOX18$^{RaOp}$ (red) separated into mobile fractions for each cell **(i)** and for each sample **(ii)**. **(iii)** Quantification of the average MSD for mobile fractions for each cell is shown as area under the curve of the MSD. Values for the mean ± s.e.m. are shown. HALO-SOX18 n = 14 and HALO-SOX18$^{RaOp}$ n = 18 (N = 3). t-test (two-tailed, unpaired). **** P <0.0001.

**(C)** The average diffusion coefficient histogram for each cell for HALO-SOX18 (black), with the immobile to mobile threshold ($Log_{10}D$ = -1.5) shown by a pink line.

**(D)** The average diffusion coefficient histogram for each cell for HALO-SOX18$^{RaOp}$ (red), with the immobile to mobile threshold ($Log_{10}D$ = -1.5) shown by a pink line.

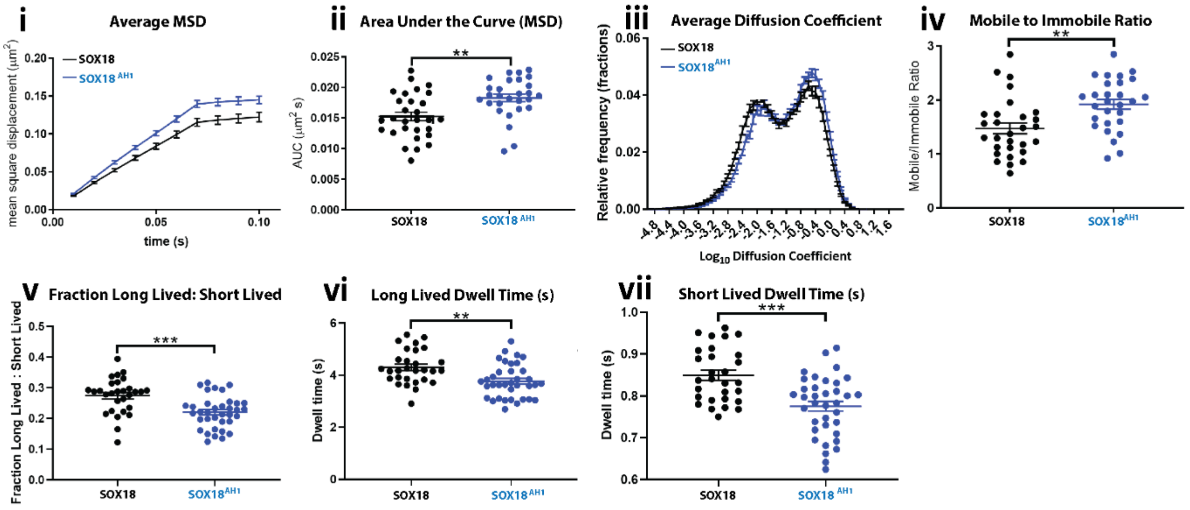


**Figure S4. A DNA-binding mutant (SOX18$^{AH1}$) shows the opposite phenotype to SOX18$^{RaOp}$.**

Quantification of the dynamics of HALO-SOX18 (black) and HALO-SOX18$^{AH1}$ (blue). **Top row:** quantification of fast single molecule tracking (SMT) data (20 ms acquisition for 6000 frames) represented by **(i)** the average mean square displacement (MSD; µm$^2$s), **(ii)** the area under the curve (AUC) of the average MSD for each cell (µm$^2$s), **(iii)** the diffusion coefficient histogram for all cells (µm$^2$s-1) and **(iv)** the mobile to immobile ratio for each cell. The threshold used to classify molecules as either mobile or immobile is $Log_{10}D$ = -1.5. Values for the mean ± s.e.m. are shown. n = 29 for HALO-SOX18 and n = 28 for HALO-SOX18$^{AH1}$ (N = 3). t-test (two-tailed, unpaired). ** P<0.01. Average number of trajectories obtained are 2713 for HALO-SOX18 and 1794 for HALO-SOX18$^{AH1}$. **Bottom row:** quantification of slow SMT data (500 ms acquisition for 500 frames) showing **(v)** the fraction of long-lived to short-lived immobile events, and dwell times of **(vi)** long-lived and **(vii)** short-lived immobile

events (s). Values for the mean ± s.e.m. are shown. n = 29 for HALO-SOX18 and n = 36 for HALO-SOX18$^{AH1}$ (N = 3). Mann-Whitney U-test (two-tailed, unpaired). ** P<0.01, *** P<0.001.

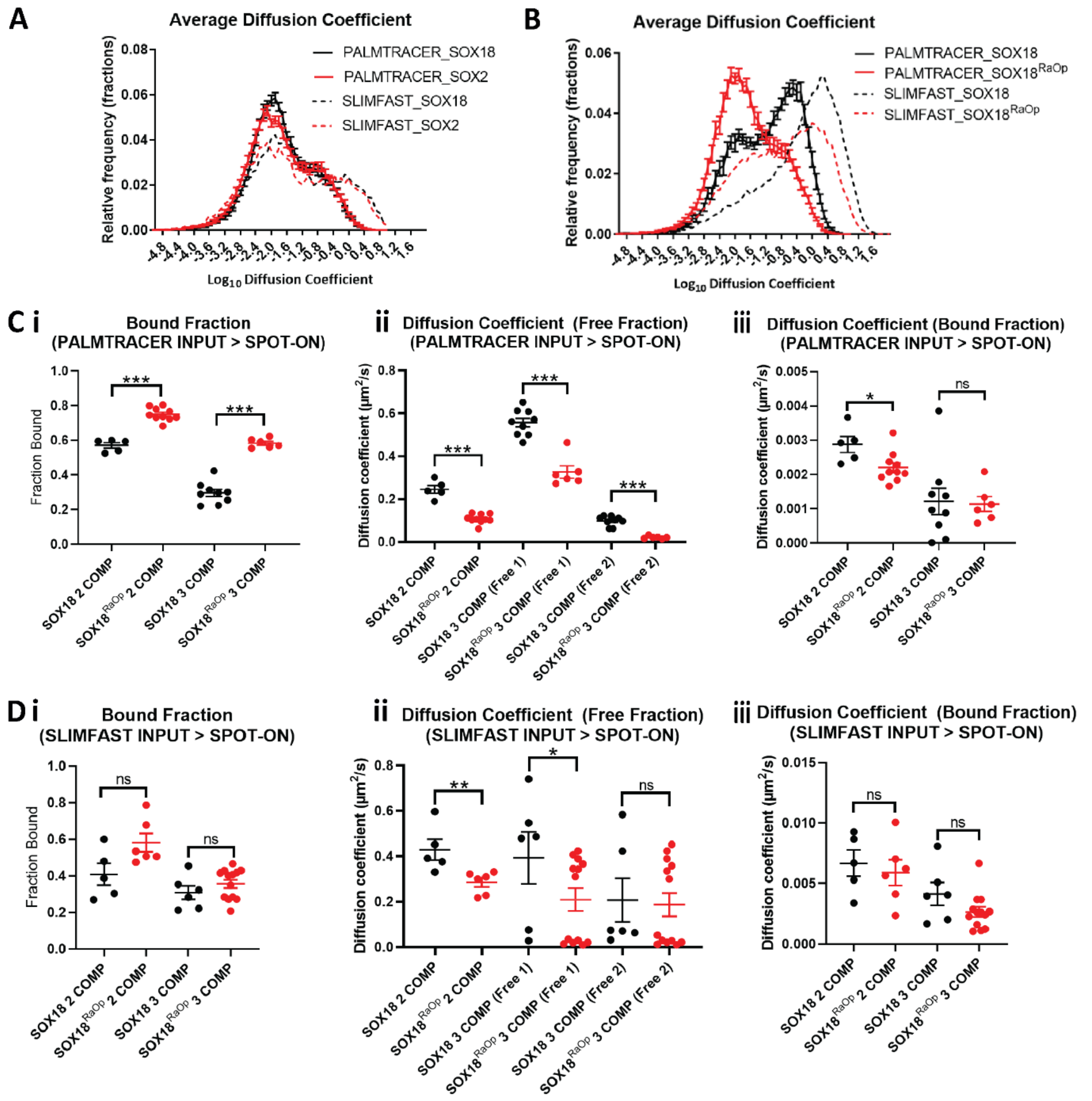


**Figure S5. Different SMT analysis software give different diffusion coefficient and chromatin-binding fraction estimates, however the trend between SOX18 and SOX18$^{RaOp}$ is reproducible.**

**(A)** Diffusion coefficient histogram for all cells (µm$^2$s-1) for HALO-SOX18 (black) and HALO-SOX2 (red) obtained using PalmTracer (full line) and SLIMfast (dashed line) analysis pipelines. n = 13 for HALO-SOX18 and n = 13 for HALO-SOX2 (N = 3).

**(B)** Diffusion coefficient histogram for all cells (µm$^2$s-1) for HALO-SOX18 (black) and HALO-SOX18$^{RaOp}$ (red) obtained using PalmTracer (full line) and SLIMfast (dashed line) analysis pipelines. n = 11 for HALO-SOX18 and n = 18 for HALO-SOX18$^{RaOp}$ (N = 3).

**(C)** Spot-On analysis (2 and 3-component fits) of HALO-SOX18 (black) and HALO-SOX18$^{RaOp}$ (red) using PalmTracer results as input. **(i)** Estimation of the bound fraction. **(ii)** Average diffusion coefficients for the unbound fraction. **(iii)** Average diffusion coefficients for the bound fraction. Values for the mean ± s.e.m. are shown. Two component analysis: n = 5 for HALO-SOX18 and n = 10 for HALO-SOX18$^{RaOp.}$ Three component analysis: n = 9 for HALO-SOX18 and n = 6 for HALO-SOX18$^{RaOp}$ (N = 3). Pairwise comparisons were assessed using a Mann-Whitney U-test (two-tailed, unpaired). * P<0.05, *** P<0.001, ns = non-significant (P>0.05).

**(D)** Spot-On analysis (2 and 3-component fits) of HALO-SOX18 (black) and HALO-SOX18$^{RaOp}$ (red) using SLIMfast results as input. **(i)** Estimation of the bound fraction. **(ii)** Average diffusion coefficients for the unbound fraction. **(iii)** Average diffusion coefficients for the bound fraction. Values for the mean ± s.e.m. are shown. Two component analysis: n = 5 for HALO-SOX18 and n = 6 for HALO-SOX18$^{RaOp.}$ Three component analysis: n = 6 for HALO-SOX18 and n = 13 for HALO-SOX18$^{RaOp}$ (N = 3). Pairwise comparisons were assessed using a Mann-Whitney U-test (two-tailed, unpaired). * P<0.05, ** P<0.01, ns = non-significant (P>0.05).

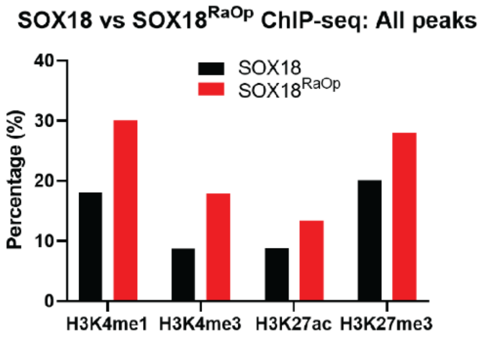


**Figure S6. SOX18$^{RaOp}$ binds to more sites associated with active histone marks than SOX18.**

**(A)** Overlay of myc-SOX18 (black) and myc-SOX18$^{RaOp}$ (red) ChIP-seq peaks in HeLa cells with active (H3Kme1, H3K4me3 and H3K27ac) and repressive (H3K27me3) histone marks. Percentage (%) refers to the percentage of histone ChIP-seq peaks that overlap with SOX18 ChIP-seq peaks by at least 50 % (in base-pairs). Histone mark datasets were obtained from ENCODE consortium (HeLa cells).

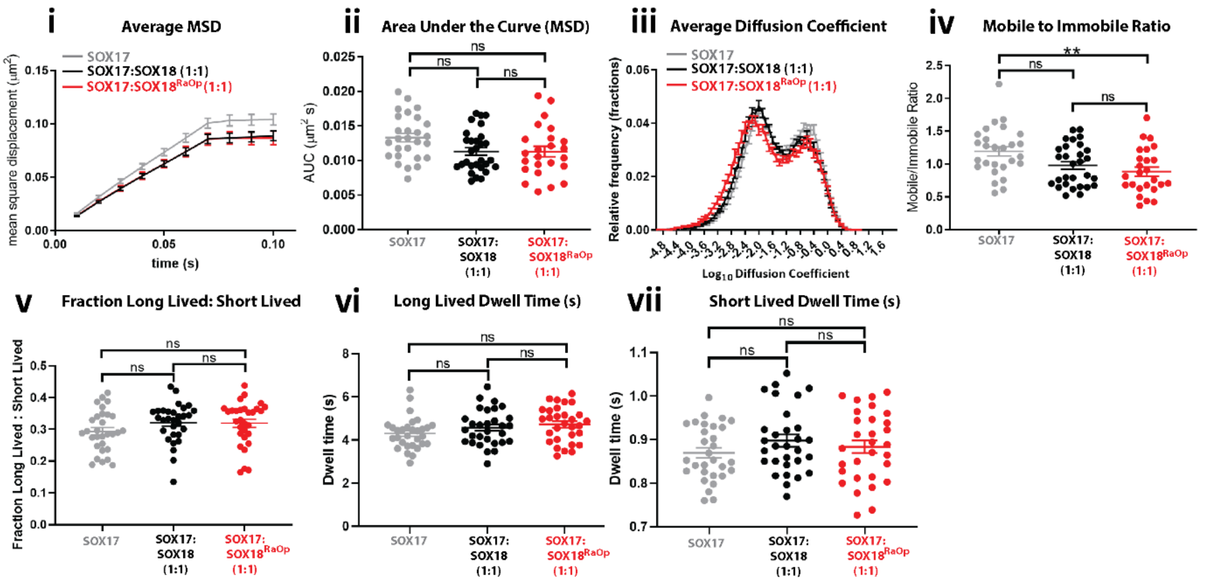


**Figures S7. SOX18$^{RaOp}$ recruits SOXF member SOX17 in a similar fashion to wild-type SOX18.**

Quantification of the dynamics of HALO-SOX17 (grey), HALO-SOX17 with untagged SOX18 in a 1:1 ratio (black) and HALO-SOX17 with untagged SOX18$^{RaOp}$ in a 1:1 ratio (red). **Top row:** quantification of fast single molecule tracking (SMT) data (20 ms acquisition for 6000 frames) represented by **(i)** the average mean square displacement (MSD; µm$^2$s), **(ii)** the area under the curve (AUC) of the average MSD for each cell (µm$^2$s), **(iii)** the diffusion coefficient histogram for all cells (µm$^2$s-1) and **(iv)** the mobile to immobile ratio for each cell. The threshold used to classify molecules as either mobile or immobile is $Log_{10}D$ = -1.5. Values for the mean ± s.e.m. are shown. Average number of trajectories obtained are 1879 for HALO-SOX17, 1863 for HALO-SOX17:SOX18 (1:1) and 1695 for HALO-SOX17:SOX18$^{RaOp}$ (1:1). n = 27 for HALO-SOX17, n = 29 for HALO-SOX17:SOX18 (1:1) and n = 24 for HALO-SOX17:SOX18$^{RaOp}$ (1:1) (N = 3). Statistical significance was determined by a Tukey post-hoc test. ** P<0.01, ns = non-significant (P>0.05). **Bottom row:** quantification of slow SMT data (500 ms acquisition for 500 frames) showing **(v)** the fraction of long-lived to short-lived immobile events and dwell times of **(vi)** long-lived and **(vii)** short-lived immobile events (s). Values for the mean ± s.e.m. are shown. n = 30 for HALO-SOX17, n = 30 for HALO-SOX17:SOX18 (1:1) and n = 30 for HALO-SOX17:SOX18$^{RaOp}$ (1:1) (N = 3). Statistical significance was determined by a Tukey post-hoc test. ns = non-significant (P>0.05).

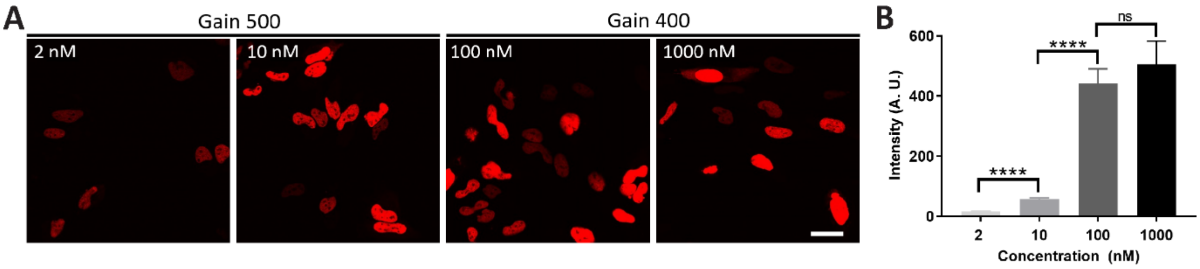


**Figure S8. JF549 dye titration to identify an optimum concentration for saturating HALO-SOX18 molecules for use in number and brightness (N&B) and cross-raster image correlation spectroscopy (cRICS) experiments.**

**(A)** Confocal images showing HeLa cells transfected with HALO-SOX18 and exposed to a titration of JF549 dye.

**(B)** Titration of JF549 dye shows that HALO-SOX18 becomes saturated with dye at approximately 1000 nM, with no significant change in intensity observed after 100 nM. Data was log transformed for ANOVA analysis, raw data is displayed. Statistical significance was determined by a Tukey post-hoc test. **** P<0.0001, ns = non-significant (P>0.05).

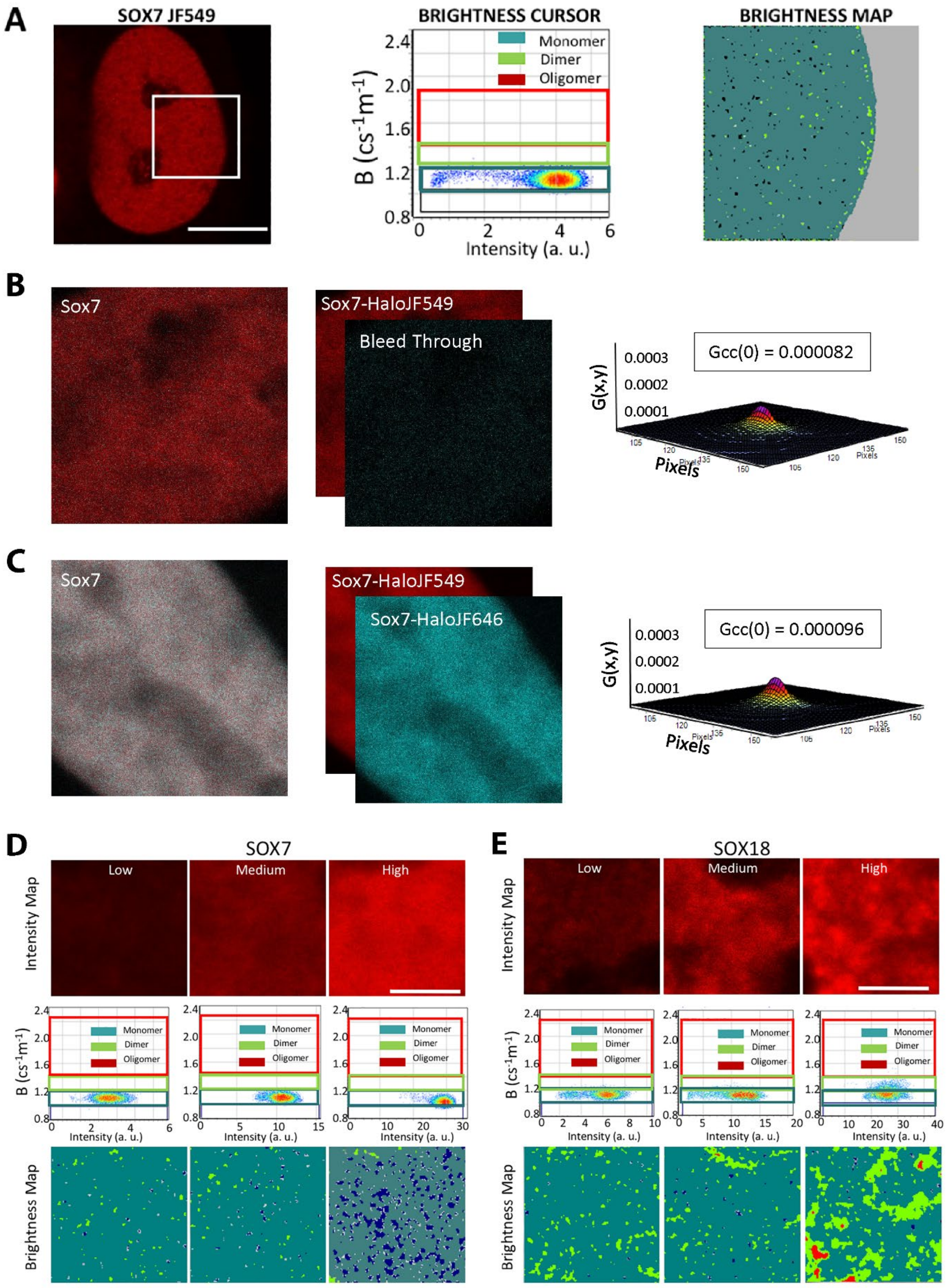


**Figure S9. SOX7 is used to calibrate monomeric molecular brightness for number and brightness (N&B) analysis and quantify a bleed-through correction for cross-raster image correlation spectroscopy (cRICS).**

**(A)** SOX7 does not form homodimers and therefore has been used as a control to calibrate the brightness for single HALO-JF549 molecules in number and brightness (N&B) analyses. **(Left)** HeLa cells are transfected with HALO-SOX7 and exposed to 1 µM of JF549 dye. The selection area on which N&B is performed is outlined by a white square. Scale bar = 10 µm. **(Middle)** Brightness histogram of HALO-SOX7 shows the monomeric brightness cursor centered at B = 1.12. The brightness cursor showing the separation of SOX7 into different oligomeric states based on brightness. As SOX7 is monomeric, the monomer cursor (dark green) for all experiments is set based on SOX7 brightness. The homodimer cursor (light green) is of the same width and placed directly above the monomer cursor, and the higher order oligomer (three or more molecules in a complex) is placed so that it contains all brightness values above the homodimer cursor. An absence of molecules is shown by a dark blue cursor directly below the monomer cursor. **(Right)** Brightness maps indicate the oligomeric distribution of HALO-SOX7, with monomers/heterodimers being represented as dark green pixels, “homodimers” as light green pixels, higher order oligomers as red pixels and an absence of molecules as dark blue pixels.

**(B) (Left):** HeLa cells were transfected with HALO-SOX7 and exposed to 500 µM of JF549 HaloTag dye. **(Middle):** Cross-correlation was performed between the JF549 and JF646 channels to quantify the amount of bleed-through from the JF549 channel into the JF646 channel. **(Right):** The 3-dimentional cRICS function for the cross-correlation between these channels showing the level of bleed-through.

**(C) (Left):** HeLa cells were transfected with HALO-SOX7 and exposed to 500 µM of JF549 and JF646 HaloTag dye, with the merging of the JF549 and JF646 channels shown. **(Middle):** The individual JF549 and JF646 channels are shown. **(Right):** The 3-dimentional cRICS function for the cross-correlation between these channels. This value is then normalized according to the bleed-through quantified in (B).

**(D)** N&B analysis performed on HALO-SOX7 using selected HeLa cells with different HALO-SOX7 concentrations. **(Top row)** An example nucleus of a cell with low HALO-SOX7 expression is shown on the left, medium expression in the middle, and high expression on the right. **(Middle row)** The brightness cursor showing the separation of HALO-SOX7 into different oligomeric states based on brightness. As SOX7 is monomeric, the monomer cursor (dark green) is set based on HALO-SOX7 brightness. The homodimer cursor (light green) is of the same width and placed directly above the monomer cursor, and the higher order oligomer (three or more molecules in a complex) is placed so that it contains all brightness values above the homodimer cursor. An absence of molecules is shown by a dark blue cursor directly below the monomer cursor. **(Bottom row)** Brightness maps indicate the oligomeric distribution of HALO-SOX7, with monomers/heterodimers being represented as dark green pixels, “homodimers” as light green pixels and an absence of molecules as dark blue pixels.

**(E)** N&B analysis performed on HALO-SOX18 using selected HeLa cells with different HALO-SOX18 concentrations. **(Top row)** An example nucleus of a cell with low HALO-SOX18 expression is shown on the left, medium expression in the middle, and high expression on the right. **(Middle row)** The brightness cursor showing the separation of SOX18 into different oligomeric states based on brightness.

The cursors are set based on the brightness values obtained for the HALO-SOX7 monomeric control. The monomer cursors are shown in dark green, the homodimer cursor in light green, the higher order oligomer cursor in red, and an absence of molecules in dark blue. **(Bottom row)** Brightness maps indicate the oligomeric distribution of HALO-SOX18, with monomers/heterodimers being represented as dark green pixels, homodimers as light green pixels, higher-order oligomers as red pixels and an absence of molecules as dark blue pixels. (Scale bar = 5 μm).

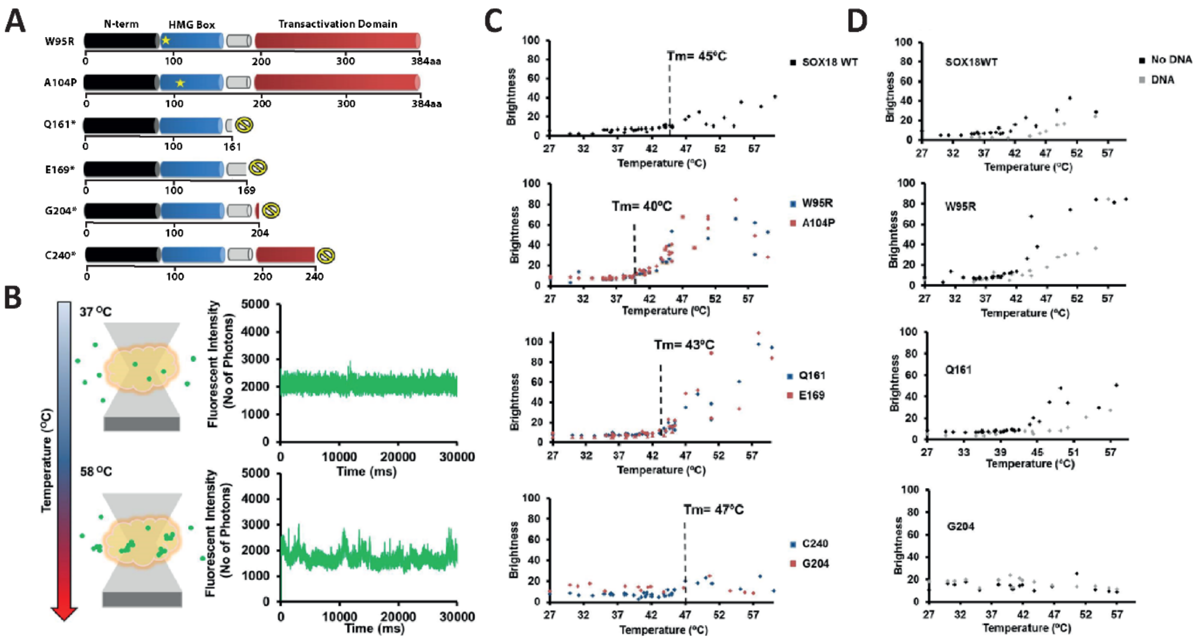


**Figure S10. Different mutations within SOX18 alters its likelihood to form homodimers and its chromatin-binding stability.**

**(A)** Schematic showing the protein domains of SOX18 and various recessive (W95R and A104P) and dominant-negative (Q161*, E169*, G204* and C240*) human mutations. A star indicates a point mutation, and a yellow 'no' symbol denotes premature truncations. SOX18 contains an N-terminal domain (black), followed by a DNA-binding and bending HMG domain (blue), a homodimerization domain (white) and a C-terminal transactivation domain (red). The most similar mutants to murine SOX18$^{RaOp}$ assessed here are human orthologs G204* and C240*, due to the truncation occurring within the transcriptional activation domain thus generating the longest non-functional protein form. Further, C240* has the most severe phenotype in humans.

**(B)** Schematic showing the fluorescence fluctuation single molecule assay performed to identify the degree of SOX18 aggregation at different temperatures (37 °C and 58 °C) which assesses the level of protein stability. Aggregation is shown by an increase in the fluctuation of fluorescence intensity indicating that multiple proteins are passing through together (bottom). Proteins with higher stabilities will require higher temperatures before they become aggregated.

**(C)** Brightness assay assessing protein stability of SOX18 and various SOX18 mutants determined by the temperature at which they become aggregated (dotted line).

**(D)** Brightness assay assessing protein stability of SOX18 and various SOX18 mutants in the presence (grey) and absence of DNA (black).

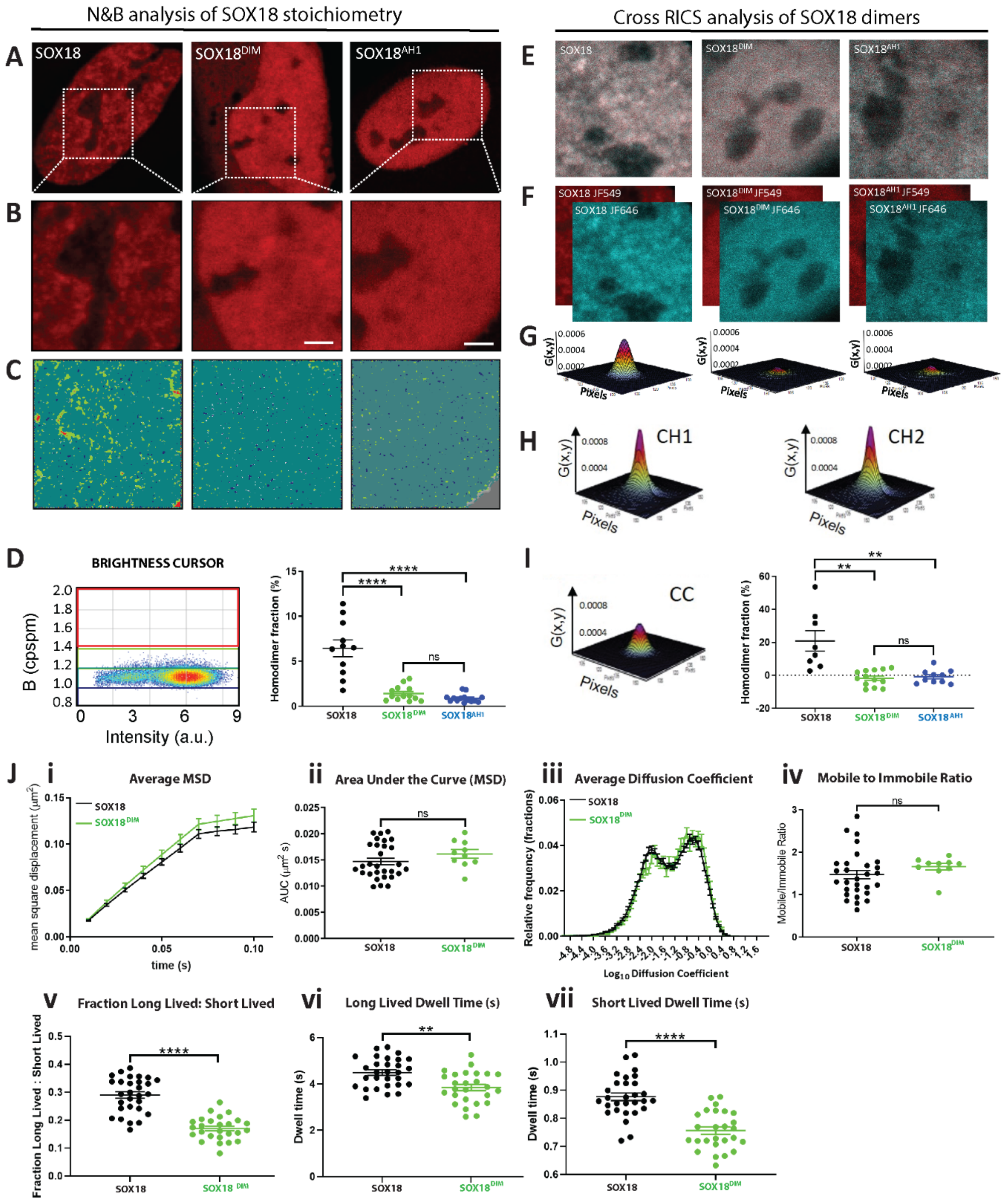


**Figure S11. SOX18 forms homodimers on the chromatin via a DNA-dependent cooperative mechanism.**

**(A-D)** Number and brightness (N&B) analysis of the oligomeric distribution of HALO-SOX18, HALO-$SOX18^{AH1}$ and HALO-$SOX18^{DIM}$ in the nucleus of HeLa cells.

**(A)** HeLa cells are transfected with **(left)** HALO-SOX18, **(middle)** HALO-$SOX18^{DIM}$ or **(right)** HALO-$SOX18^{AH1}$ and exposed to 1 nM of JF549 HaloTag dye.

**(B)** The selection area on which N&B analysis was performed. Scale bar = 2 µm.

**(C)** The brightness map showing the distribution of SOX18 monomers (dark green), homodimers (light green) and higher order oligomers (red). An absence of tagged molecules is shown in dark blue.

**(D)** **(Left)** Intensity versus brightness scatterplot of the N&B data acquisition presented for HALO-SOX18. SOX18 monomers are outlined by a dark green selection box, SOX18 homodimers by a light green selection box and SOX18 higher order oligomers (3 or more SOX18 molecules in a complex) by a red selection box. The position of the selection boxes was determined based on a HALO-SOX7 monomeric control (shown in Fig. S9A-D). **(Right)** The percentage of homodimers for HALO-SOX18 (black), HALO-$SOX18^{DIM}$ (green) and HALO-$SOX18^{AH1}$ (blue). Values for the mean ± s.e.m. are shown. HALO-SOX18 n = 11, HALO-$SOX18^{DIM}$ n = 13 and HALO-$SOX18^{AH1}$ n = 14. Data was log transformed for ANOVA analysis, raw data is displayed. Statistical significance was determined by a Tukey post-hoc test. **** P<0.0001, ns = non-significant.

**(E-G)** Cross-raster image correlation spectroscopy (cRICS) analysis validating that wild-type SOX18 forms homodimers, whereas $SOX18^{DIM}$ and $SOX18^{AH1}$ does not.

**(E)** HeLa cells were transfected with **(left)** HALO-SOX18, **(middle)** HALO-$SOX18^{DIM}$ or **(right)** HALO-$SOX18^{AH1}$ and exposed to 500 nM of JF549 and 500 nM of JF646 HaloTag dye, with the merging of the JF549 and JF646 channels shown.

**(F)** The individual JF549 and JF646 channels are shown.

**(G)** The 3D cRICS correlation profiles obtained for **(left)** HALO-SOX18, **(middle)** HALO-$SOX18^{DIM}$ and **(right)** HALO-$SOX18^{AH1}$ showing high correlation for SOX18 (forms homodimers) and low correlations for $SOX18^{DIM}$ and $SOX18^{AH1}$ (does not form homodimers).

**(H)** The 3D RICS correlation profile obtained for HALO-SOX18 molecules detected in **(left)** the JF549 channel (CH1) versus **(right)** the JF646 channel (CH2) that were fit to a two-component 3D diffusion model.

**(I)** **(Left)** The 3D cross-RICS correlation profile obtained for HALO-SOX18 dimers detected in both the JF549 and JF646 channels (CC), that was fit to a 1-component 3D diffusion model channel, enabling **(right)** the quantification of the fraction of homodimers/oligomers for HALO-SOX18, HALO-$SOX18^{DIM}$ and HALO-$SOX18^{AH1}$. Values for the mean ± s.e.m. are shown. HALO-SOX18 n = 8, HALO-$SOX18^{DIM}$ n = 13 and HALO-$SOX18^{AH1}$ n = 10. Statistical significance was determined by Kruskal-Wallis analysis. ** P<0.01, ns = non-significant.

**(J)** Quantification of the dynamics of HALO-SOX18 (black) and HALO-SOX18$^{DIM}$ (green). **Top row:** quantification of fast single molecule tracking (SMT) data (20 ms acquisition for 6000 frames) represented by **(i)** the average mean square displacement (MSD; μm$^2$s), **(ii)** the area under the curve (AUC) of the average MSD for each cell (μm$^2$s), **(iii)** the diffusion coefficient histogram for all cells (μm$^2$s-1) and **(iv)** the mobile to immobile ratio for each cell. The threshold used to classify molecules as either mobile or immobile is $Log_{10}D$ = -1.5. Average number of trajectories obtained are 2379 for HALO-SOX18 and 1356 for HALO-SOX18$^{DIM}$. n = 29 for HALO-SOX18 and n = 10 for HALO-SOX18$^{DIM}$ (N = 3). t-test (two-tailed, unpaired). ns = non-significant. **Bottom row:** quantification of slow SMT data (500 ms acquisition for 500 frames) showing **(v)** the fraction of long-lived to short-lived immobile events, and dwell times of **(vi)** long-lived and **(vii)** short-lived immobile events (s). Values for the mean ± s.e.m. are shown. n = 29 for HALO-SOX18 and n = 26 for HALO-SOX18$^{DIM}$ (N = 3). Mann Whitney U-test (two-tailed, unpaired). ** P<0.01, **** P<0.0001.

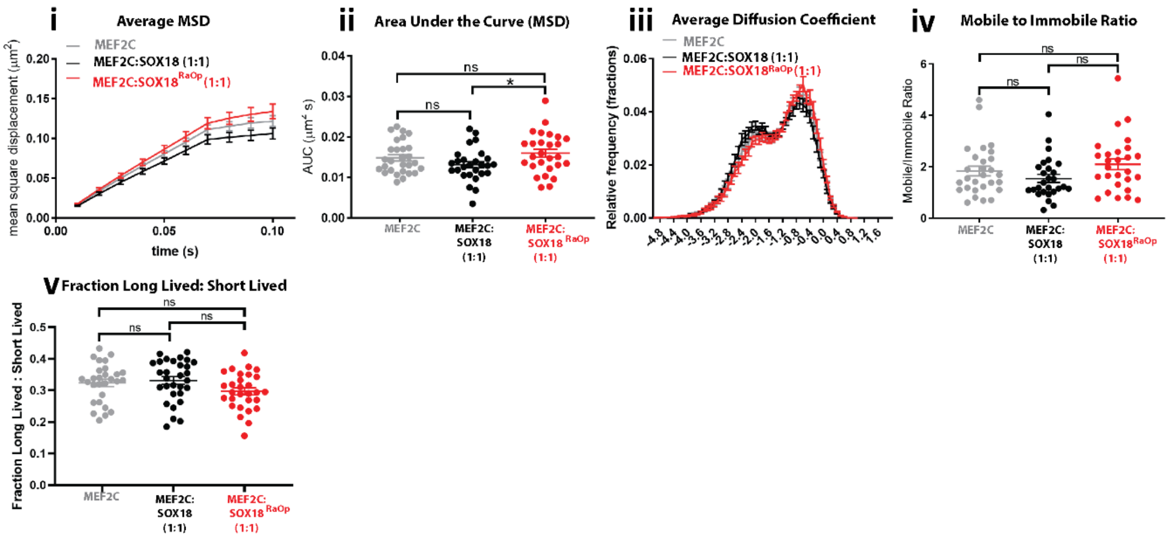


**Figure S12. SOX18$^{RaOp}$ causes an increase in MEF2C diffusion compared to SOX18.**

Quantification of the dynamics of HALO-MEF2C (grey), HALO-MEF2C with untagged SOX18 in a 1:1 ratio (black) and HALO-MEF2C with untagged SOX18$^{RaOp}$ in a 1:1 ratio (red). **(Top row):** quantification of fast single molecule tracking (SMT) data (20 ms acquisition for 6000 frames) represented by **(i)** the average mean square displacement (MSD; μm$^2$s), **(ii)** the area under the curve (AUC) of the average MSD for each cell (μm$^2$s), **(iii)** the diffusion coefficient histogram for all cells (μm$^2$s-1) and **(iv)** the mobile to immobile ratio for each cell. The threshold used to classify molecules as either mobile or immobile is $Log_{10}D$ = -1.5. Values for the mean ± s.e.m. are shown. The average number of trajectories obtained are 2275 for HALO-MEF2C, 2408 for HALO-MEF2C:SOX18 (1:1) and 1737 for HALO-MEF2C:SOX18$^{RaOp}$ (1:1). n = 28 for HALO-MEF2C, n = 27 for HALO-MEF2C:SOX18 (1:1) and n = 27 for HALO-MEF2C:SOX18$^{RaOp}$ (1:1) (N = 3). Immobile to immobile ratio data was log transformed for ANOVA analysis, raw data is displayed. Statistical significance was determined by a Tukey post-hoc test. * P<0.05, ns = non-significant (P>0.05). **(Bottom row):** quantification of slow SMT data (500 ms acquisition for 500 frames) showing **(v)** the fraction of long-lived to short-lived immobile events. Values

for the mean ± s.e.m. are shown. n = 28 for HALO-MEF2C, n = 30 for HALO-MEF2C:SOX18 (1:1) and n = 29 for HALO-MEF2C:SOX18$^{RaOp}$ (1:1) (N = 3). Statistical significance was determined by a Tukey post-hoc test. ns = non-significant (P>0.05).

## SUPPLEMENTARY TABLES

**Table S1 containing plasmid information has been attached externally as an excel sheet due to size.**

| | DiffCo (bound) | DiffCo (unbound) | bound % |
|---|---|---|---|
| PalmTracer (threshold = -1.5) | | | |
| SOX18 | 0.02 | 0.43 | 34% |
| $SOX18^{RaOp}$ | 0.02 | 0.27 | 58% |
| SLIMfast (threshold = -1.5) | | | |
| SOX18 | 0.01 | 2.54 | 14% |
| $SOX18^{RaOp}$ | 0.01 | 1.51 | 27% |
| SLIMfast (threshold = -1) | | | |
| SOX18 | 0.1 | 3.4 | 37% |
| $SOX18^{RaOp}$ | 0.1 | 2.5 | 57% |
| Spot-On (using PalmTracer input, 2-COMP) | | | |
| SOX18 | 0.003 | 0.24 | 57% |
| $SOX18^{RaOp}$ | 0.002 | 0.11 | 75% |
| Spot-On (using PalmTracer input, 3-COMP) | | | |
| SOX18 | 0.001 | 0.56; 0.1 | 30% |
| $SOX18^{RaOp}$ | 0.001 | 0.33; 0.02 | 58% |
| Spot-On (using SLIMfast input, 2-COMP) | | | |
| SOX18 | 0.007 | 0.43 | 41% |
| $SOX18^{RaOp}$ | 0.006 | 0.28 | 58% |
| Spot-On (using SLIMfast input 3-COMP) | | | |
| SOX18 | 0.004 | 0.39; 0.21 | 31% |
| $SOX18^{RaOp}$ | 0.003 | 0.21; 0.19 | 36% |

**Table S2.** Diffusion coefficients obtained for the bound and unbound fractions, and the percentage of molecules that are bound, using the same SOX18 and $SOX18^{RaOp}$ datasets, as estimated using different SMT analysis software. Threshold used to classify mobile and immobile molecules is $Log_{10}D$ = -1.5 for PalmTracer as derived from Equation 2. This threshold, as well as an adjusted threshold of $Log_{10}D$ = -1 to account for the shift in the data, was used for SLIMfast analysis. Spot-On analysis was performed using either PalmTracer or SLIMfast analysis files as input, and using both 2- and 3-component analysis.

**Table S3 containing the top 30 enriched motifs for each ChIP-seq condition has been attached externally due to size.**

**SUPPLEMENTARY VIDEOS**

Examples of single molecule tracking movies acquired for each condition using fast and slow tracking methods (listed below) can be accessed using the DataDryad link: https://doi.org/10.5061/dryad.8w9ghx3n7

Video S1 – SOX18 VS SOX18RaOp (FAST TRACKING)
Video S2 – SOX18 VS SOX18RaOp (SLOW TRACKING)
Video S3 – SOX18 WITH SOX18RaOp (FAST TRACKING)
Video S4 – SOX18 WITH SOX18RaOp (SLOW TRACKING)
Video S5 – SOX7 (FAST TRACKING)
Video S6 – SOX7 (SLOW TRACKING)
Video S7 – SOX17 (FAST TRACKING)
Video S8 – SOX17 (SLOW TRACKING)
Video S9 – SOX18DIM (FAST TRACKING)
Video S10 – SOX18DIM (SLOW TRACKING)
Video S11 – SOX18AH1 (FAST TRACKING)
Video S12 – SOX18AH1 (SLOW TRACKING)
Video S13 – MEF2C (SLOW TRACKING)
Video S14 – MEF2C (FAST TRACKING)
